# Pediatric Hospitalizations and Emergency Department Visits Related to Mental Health Conditions and Self-Harm

**DOI:** 10.1001/jamanetworkopen.2024.41874

**Published:** 2024-10-29

**Authors:** Zaba Valtuille, Vincent Trebossen, Naim Ouldali, Aurélie Bourmaud, Coralie Gandré, Camille Aupiais, Sandrine Katsahian, Richard Delorme, Hugo Peyre, Florentia Kaguelidou

**Affiliations:** 1 Center of Clinical Investigations, INSERM CIC1426, Robert Debré University Hospital, Assistance Publique–Hôpitaux de Paris (AP-HP) Nord, Paris, France; 2 URP7323 Perinatal and Pediatric Pharmacology and Therapeutic Assessment, Paris Cité University, Paris, France; 3 Department of Child and Adolescent Psychiatry, Robert Debré University Hospital, AP-HP Nord, Paris Cité University, Paris, France; 4 Department of General Pediatrics, Pediatric Infectious Disease and Internal Medicine, Robert Debré University Hospital, AP-HP Nord, Paris Cité University, Paris, France; 5 Clinical Epidemiology Unit, INSERM CIC1426, Robert Debré University Hospital, AP-HP Nord, Paris, France; 6 UMR-S 1123, Épidémiologie Clinique et Évaluation Économique Appliqué aux Populations Vulnérables, Paris Cité University, Paris, France; 7 Department of Medical Informatics, Robert Debré University Hospital, AP-HP Nord, Paris, France; 8 Institute for Research and Information in Health Economics, Paris, France; 9 Department of Pediatric Emergency Care, AP-HP Hôpital Jean-Verdier, Seine- Saint-Denis, France; 10 Unité de Recherche Clinique, AP-HP Hôpital Européen Georges Pompidou, AP-HP Centre, Paris, France; 11 HeKA Team, INRIA, INSERM CIC1218-Epidémiologie Clinique, Université Paris Cité, Paris, France; 12 Human Genetics and Cognitive Functions, Institut Pasteur, Paris, France; 13 CESP, INSERM U1178, Centre de Recherche en Épidémiologie et Santé des Populations, Villejuif, France

## Abstract

**Importance:**

Mental disorders among the pediatric population are a major area of public health concern. Little is known regarding changes in pediatric hospital resource use related to mental health (MH) long after the onset of the COVID-19 pandemic in March 2020.

**Objective:**

To assess rates and trends of hospitalizations and emergency department (ED) visits related to MH and self-harm (SH) among children before and during the 3 years following the pandemic onset.

**Design, Setting, and Participants:**

This cross-sectional study used national hospital data. The study sample included all MH- and SH-related hospitalizations and ED visits among children aged 6 to 17 years in France between January 1, 2016, to May 31, 2023.

**Main Outcomes and Measures:**

Interrupted time-series analysis of monthly rates of MH- and SH-related hospitalizations and ED visits per 100 000 children was conducted to assess changes before and every year after the pandemic onset. Rate ratios (RRs) between estimated and expected rates were calculated.

**Results:**

Overall, 583 244 hospitalizations (81.4% for MH and 18.6% for SH) and 432 725 ED visits (79.9% for MH and 20.1% for SH) were analyzed. The mean (SD) age of the children was 13.7 (2.9) and 14.8 (1.7) years for MH-related and SH-related hospitalizations, respectively, and 14.2 (2.6) and 14.6 (2.1) years for MH-related and SH-related ED visits, respectively. For MH-related hospitalizations, 52.6% were female and 47.4% were male; for SH-related hospitalizations, 83.1% were female and 16.9% were male. For MH-related ED visits, 62.8% were female and 37.2% were male; for SH-related ED visits, 77.4% were female and 22.6% were male. Before the pandemic, an increasing trend in all monthly rates, except that of MH-related hospitalizations, was observed. After an immediate decrease in hospitalization and ED visit rates during the initial pandemic period (March 1 to May 31, 2020), trends increased in the first 2 years following the pandemic onset and decreased thereafter. Overall, rates of MH-related hospitalizations and ED visits exceeded expected rates in only the second year after the pandemic onset, with increases of 6.0% (RR, 1.06 [95% CI, 1.05-1.06]) and 5.0% (RR, 1.05 [95% CI, 1.04-1.05]), respectively. However, rates of hospitalizations and ED visits for behavioral syndromes (mainly eating and sleeping disorders) persistently exceeded expected rates, with increases of 29.0% (RR, 1.29 [95% CI, 1.25-1.34]) and 26.0% (RR, 1.26 [95% CI, 1.21-1.31]) in the third year, respectively. Likewise, rates of SH-related hospitalizations and ED visits persistently rose above expected rates, with increases of 29.0% (RR, 1.29 [95% CI, 1.26-1.32]) and 43.0% (RR, 1.43 [95% CI, 1.40-1.47]) in the third year, respectively.

**Conclusions and Relevance:**

In this cross-sectional study, persistent increases in the use of hospital resources to treat eating and sleeping disorders and intentional SH among pediatric patients were observed long after the onset of the COVID-19 pandemic. These findings warrant future research to identify persistent stress factors in children.

## Key Points

**Question:**

Did monthly rates of hospitalizations and emergency department (ED) visits related to mental health (MH) and self-harm (SH) among children change following the onset of the COVID-19 pandemic?

**Findings:**

This cross-sectional study analyzed 583 244 hospitalizations and 432 725 ED visits from 2016 to 2023. Rates of hospitalizations and ED visits related to MH exceeded expected rates in only the second year after the pandemic onset (6% and 5%, respectively), whereas those related to SH persistently increased above expected rates after the pandemic onset (28% and 43% in the third year, respectively).

**Meaning:**

These findings suggest that the strain on pediatric hospital resource use related to MH disorders, and specifically to SH, persisted in the 3 years following the pandemic onset.

## Teaser

This cross-sectional study examines rates of hospitalizations and emergency department (ED) visits related to mental health and self-harm among children aged 6 to 17 years in France before and after the onset of the COVID-19 pandemic.

## Introduction

Mental disorders among the pediatric population have been pinpointed as a major health concern over the last decade.^1,2^ The unprecedented disruptions caused by the COVID-19 pandemic, including social distancing measures, socioeconomic turmoil, and fear of infection, created fertile ground for mental health (MH) disorders to thrive.^3,4,5^ Studies reported sharp increases in the prevalence of illness-related fear and depressive and anxiety symptoms among children and adolescents compared with prepandemic estimates.^6,7,8,9^ Among adolescents, the negative effects of the COVID-19 pandemic on MH appeared to be more pronounced in girls, with greater increases in depressive and anxiety symptoms compared with boys.^9,10,11^

The number of emergency department (ED) visits for suicidality among children and adolescents was already on the rise before the pandemic, and it increased substantially in the months following the pandemic outbreak worldwide, particularly among adolescent girls.^10,12,13^ Hospitalizations associated with MH conditions among children and adolescents, especially those related to acute anxiety and mood disorders, also increased shortly after the start of the pandemic.^14,15^ However, little is known about the use of MH-related inpatient and acute hospital resources long after the pandemic onset. Our study aimed to assess rates and trends of hospitalizations and ED visits related to MH and intentional SH among children before and in the 3 years following the onset of the pandemic.

## Methods

### Study Design and Setting

For this cross-sectional study, we conducted interrupted time-series analysis of data from the French hospital discharge database (Programme de Médicalisation des Systèmes d’Information [PMSI]). The PMSI comprises inpatient health claims data for individuals attending public and private health institutions on a nationwide basis.^16^ The PMSI includes exhaustive administrative information, medical procedures, and discharge diagnoses for every hospital stay. A specific dataset on the activity of the EDs of private or public health institutions (Résumé de Passage aux Urgences [RPU]) is also available. The RPU dataset comprises exhaustive medicoadministrative patient data and covers the activity of 84% to 95% of EDs in France (eMethods 1 in Supplement 1).^17^ The project protocol was approved by the French National Ethics and Scientific Committee for Health Research, Studies, and Evaluations and by the French Data Protection Authority, and it is publicly available (NCT05260866). The requirement to obtain patient informed consent was waived because the study did not involve active personal participation or any identifiable medical data. This study followed the Strengthening the Reporting of Observational Studies in Epidemiology (STROBE) reporting guideline.

### Study Sample

The study sample included all hospitalizations and ED visits related to an MH condition or SH among children aged 6 to 17 years in France (excluding overseas territories) between January 1, 2016, and May 31, 2023. Hospitalizations and ED visits for MH conditions were identified using diagnostic codes (*International Statistical Classification of Diseases, Tenth Revision* [*ICD-10*]) for mental and behavioral disorders (codes F00-F99). Hospitalizations and ED visits for SH were identified using *ICD-10* codes for intentional SH or suicide attempts (codes X60-X84). Details are provided in eMethods 2 and 3 in Supplement 1.

### Study Outcomes

The primary study outcomes were the monthly rates of MH- and SH-related hospitalizations and ED visits per 100 000 children aged 6 to 17 years. Rates were calculated using the counts of every primary outcome (numerator) and appropriate age- and sex-specific population estimates derived from the National Institute of Statistics and Economic Studies (denominator), both aggregated at a monthly level.^18^ Rates of ED visits were corrected for the annual percentage of national coverage of the RPU dataset. Secondary outcomes include monthly rates of MH- and SH-related hospitalizations and ED visits per 100 000 children by (1) principal MH condition and (2) by age (6-11 years and 12-17 years) and sex (female or male).

### Study Periods

The study involved 5 predefined time periods. These periods were as follows: before pandemic onset (January 1, 2016, to February 29, 2020), initial pandemic period (March 1 to May 31, 2020, corresponding to strict home confinement of the population), and the first (June 1, 2020, to May 31, 2021), second (June 1, 2021, to May 31, 2022), and third (June 1, 2022, to May 31, 2023) years after the pandemic onset.

### Study Measures

For each hospitalization or ED visit, the following data were retrieved: patient age and sex, socioeconomic status, duration of hospital stay (in days), principal diagnosis, means of intentional SH, and patient status (hospitalized, discharged, transferred, or deceased) at discharge. Socioeconomic status was considered low if the patient was a beneficiary of supplementary universal health coverage (Complémentaire Santé Solidaire [C2S]), which is not available for ED visits.

### Statistical Analysis

Characteristics of hospitalizations and ED visits before and after the onset of the COVID-19 pandemic were described and compared using appropriate statistical tests. The χ^2^ or Fisher exact test was used for categorical variables, and the *t* test or Mann-Whitney–Wilcoxon test was used for continuous variables.

Interrupted time-series analyses of the primary study outcomes were performed using log-linear regression models with autoregressive moving average errors, accounting for autocorrelation, seasonality, and trend before the pandemic.^19,20,21^ Seasonality was accounted for by means of a multiplicative model. In the initial pandemic period, we hypothesized an immediate change in the monthly rate of MH- and SH-related hospitalizations and ED visits (level change), whereas the change over the 3 years following the pandemic onset was expected to be progressive (trend changes) (eMethods 4 in Supplement 1). Model validity was assessed by visual inspection of correlograms and residual analyses. Estimates of level and trend changes were interpreted as percentage changes with 95% CIs. Further, regression model estimates of hospitalization and ED visit rates after the pandemic were compared with the expected rates forecasted by the model based on prepandemic data only to calculate rate ratios (RRs) with 95% CIs computed via bootstrapping (10 000 replications). All analyses were stratified by MH condition and by age and sex.

We performed several sensitivity analyses. We used (1) a log-linear regression model adjusted to the total monthly number of pediatric hospitalizations or ED visits to control for changes over time that could affect model estimates, (2) a negative binomial regression model accounting for seasonality and autocorrelation, and (3) a log-linear regression using a more stringent definition of SH-related hospitalizations and ED visits.

All statistical tests were 2-sided, and *P* < .05 was considered statistically significant. Statistical analyses were performed using R software, version 4.2.2 (R Project for Statistical Computing).

## Results

Overall, 583 244 hospitalizations (81.4% for MH and 18.6% for SH) and 432 725 ED visits (79.9% for MH and 20.1% for SH) were analyzed. The mean (SD) age of the children was 13.7 (2.9) and 14.8 (1.7) years for MH-related and SH-related hospitalizations, respectively, and 14.2 (2.6) and 14.6 (2.1) years for MH-related and SH-related ED visits, respectively. For MH-related hospitalizations, 52.6% were female and 47.4% were male; for SH-related hospitalizations, 83.1% were female and 16.9% were male. For MH-related ED visits, 62.8% were female and 37.2% were male; for SH-related ED visits, 77.4% were female and 22.6% were male. Complete demographic characteristics of the sample are provided in Table 1.

**Table 1.** Characteristics of Hospitalizations and ED Visits Related to Mental Health and Self-Harm Among Children, Before and After COVID-19 Pandemic Onset^a^

Characteristic	Mental health conditions						Self-harm					
	Hospitalizations (n = 474 999)			ED visits (n = 345 563)			Hospitalizations (n = 108 245)			ED visits (n = 87 162)		
	No. (%)		*P* value^b^	No. (%)		*P* value^b^	No. (%)		*P* value^b^	No. (%)		*P* value^b^
	Before pandemic onset (n = 272 201)	After pandemic onset (n = 202 798)		Before pandemic onset (n = 180 610)	After pandemic onset (n = 164 953)		Before pandemic onset (n = 52 361)	After pandemic onset (n = 55 884)		Before pandemic onset (n = 35 245)	After pandemic onset (n = 51 917)	
Age at hospitalization or ED visit, mean (SD), y	13.57 (2.97)	13.77 (2.83)	<.001	14.15 (2.71)	14.23 (2.53)	<.001	14.83 (1.69)	14.78 (1.65)	<.001	14.63 (2.18)	14.6 (2.01)	.11
Age group, y												
Children (aged 6-11)	62 725 (23.0)	42 318 (20.9)	<.001	30 105 (16.7)	23 572 (14.3)	<.001	1654 (3.2)	1586 (2.8)	.002	2635 (7.5)	3241 (6.2)	<.001
Adolescents (aged 12-17)	209 476 (77.0)	160 480 (79.1)		150 505 (83.3)	141 381 (85.7)		50 707 (96.8)	54 298 (97.2)		32 610 (92.5)	48 676 (93.8)	
Sex												
Female	132 778 (48.8)	117 082 (57.7)	<.001	107 989 (59.8)	109 157 (66.2)	<.001	42 336 (80.9)	47 664 (85.3)	<.001	26 289 (74.6)	41 202 (79.4)	<.001
Male	139 423 (51.2)	85 716 (42.3)		72 621 (40.2)	55 796 (33.8)		10 025 (19.1)	8220 (14.7)		8956 (25.4)	10 715 (20.6)	
Duration of hospital stay, median (IQR), d	5 (2-11)	5 (2-13)	<.001	NA	NA	NA	3 (1-7)	4 (2-7)	<.001	NA	NA	NA
Socioeconomic status												
Low	48 179 (17.7)	41 579 (20.5)	<.001	NA	NA	NA	9949 (19.0)	12 026 (21.5)	<.001	NA	NA	NA
Patient status after hospitalization or ED visit												
Hospitalized	NA	NA	NA	50 145 (27.8)	50 884 (30.9)	<.001	NA	NA	NA	24 458 (69.4)	35 708 (68.8)	<.001
Discharged	254 255 (93.4)	189 900 (93.6)	.001	123 797 (68.5)	109 966 (66.7)	<.001	44 609 (85.2)	46 800 (83.7)	<.001	9303 (26.4)	15 070 (29.0)	<.001
Transferred	16 578 (6.1)	12 259 (6.0)	.52	NA	NA	NA	7658 (14.7)	8988 (16.1)	<.001	NA	NA	NA
Deceased	47 (0.0)	37 (0.0)	.88	8 (0.0)	10 (0.0)	.66	59 (0.1)	67 (0.1)	.79	3 (0.0)	2 (0.0)	>.99
Unknown	1321 (0.5)	602 (0.3)	<.001	6660 (3.7)	4063 (2.5)	<.001	35 (0.1)	29 (0.1)	.37	1481 (4.2)	1137 (2.2)	<.001
Means of intentional self-harm^c^												
Drug self-poisoning	NA	NA	NA	NA	NA	NA	34 324 (65.6)	35 939 (64.3)	<.001	27 477 (78.0)	32 324 (62.3)	<.001
Self-poisoning by other products	NA	NA	NA	NA	NA	NA	2099 (4.0)	1791 (3.2)	<.001	94 (0.3)	110 (0.2)	.08
Violent means	NA	NA	NA	NA	NA	NA	16 868 (32.2)	19 996 (35.8)	<.001	1547 (4.4)	1542 (3.0)	.02
Other means	NA	NA	NA	NA	NA	NA	2452 (4.7)	2739 (4.9)	.10	484 (1.4)	436 (0.8)	.56
Sequelae of intentional self-harm	NA	NA	NA	NA	NA	NA	NA	NA	NA	49 (0.1)	83 (0.2)	<.001
Suicidal ideation	NA	NA	NA	NA	NA	NA	NA	NA	NA	9749 (27.7)	23 069 (44.4)	<.001

Abbreviations: ED, emergency department; NA, not applicable.

^a^ Reported as before pandemic onset (January 1, 2016, to February 29, 2020) and after pandemic onset (March 1, 2020, to May 31, 2023).

^b^ A χ^2^ test was used for age groups, sex, socioeconomic status, vital status, and means of self-harm. A *t* test was used for age. A Mann-Whitney-Wilcoxon test was used for duration of hospital stay.

^c^ Does not sum to 100%, as some hospitalizations or ED visits may have more than 1 diagnosis.

### Hospitalizations and ED Visits Related to MH Conditions

Overall, we analyzed 474 999 hospitalizations and 345 563 ED visits related to MH conditions. Compared with the prepandemic period, the proportions of children aged 12 to 17 years (77.0% vs 79.1%) and of those with low socioeconomic status (17.7% vs 20.5%) who were hospitalized for an MH-related condition increased after the onset of the pandemic. Similarly, the proportion of girls increased from before to after the pandemic onset for both MH-related hospitalizations (48.8% vs 57.7%) and ED visits (59.8% vs 66.2%) (Table 1).

**Figure 1.**
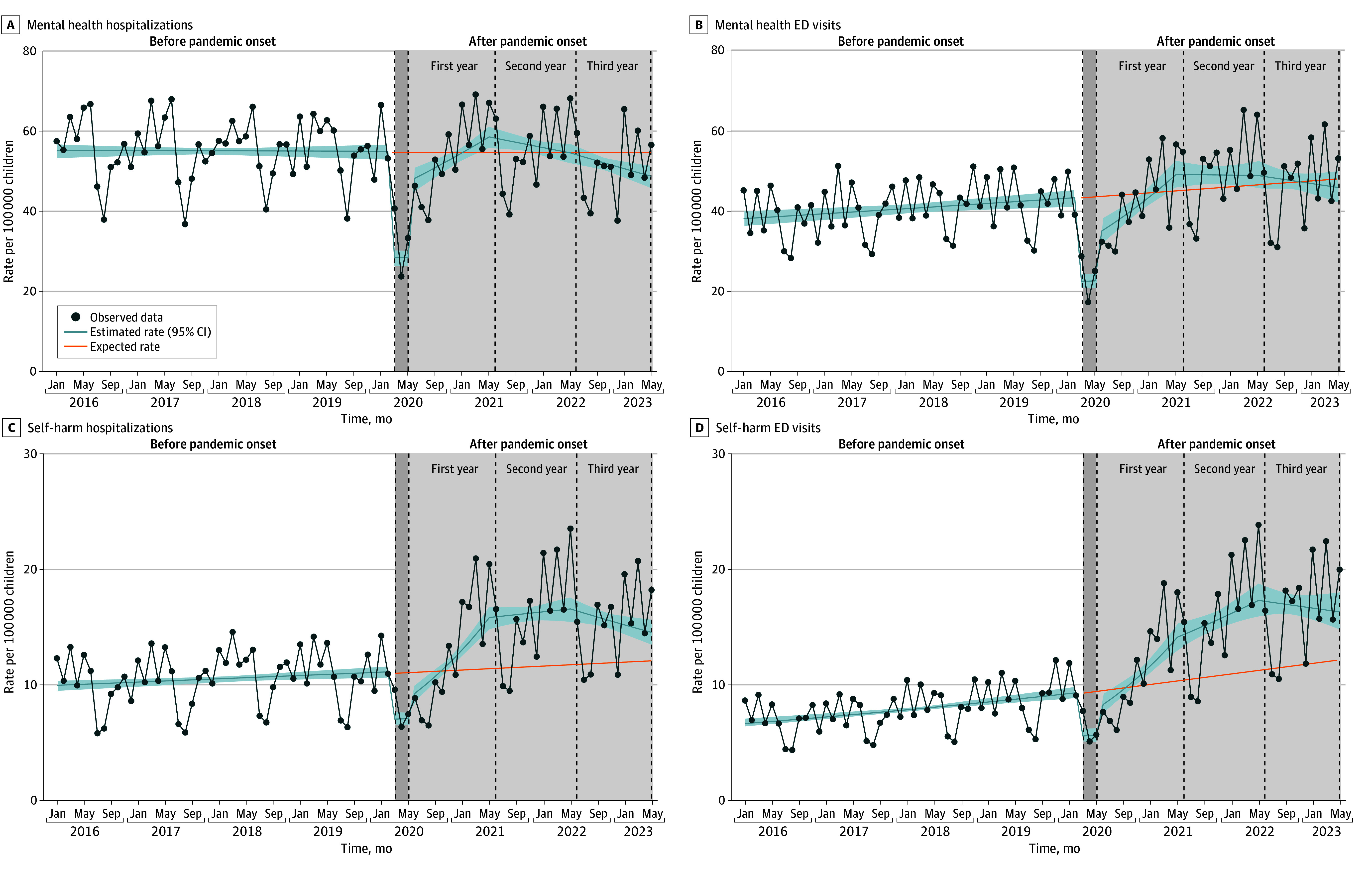
Rates of Hospitalizations and Emergency Department (ED) Visits Related to Mental Health and Self-Harm Among Children, Before and After Onset of the COVID-19 Pandemic Observed data indicate observed hospitalization or ED visit rates per 100 000 children. Rates estimated by the model include corresponding 95% CIs (indicated by shaded areas). Expected rates are based on prepandemic data. The study involved the following time periods: before pandemic onset (January 1, 2016, to February 29, 2020), initial pandemic period (March 1 to May 31, 2020), and the first (June 1, 2020, to May 31, 2021), second (June 1, 2021, to May 31, 2022), and third (June 1, 2022, to May 31, 2023) years after the pandemic onset.

**Figure 2.**
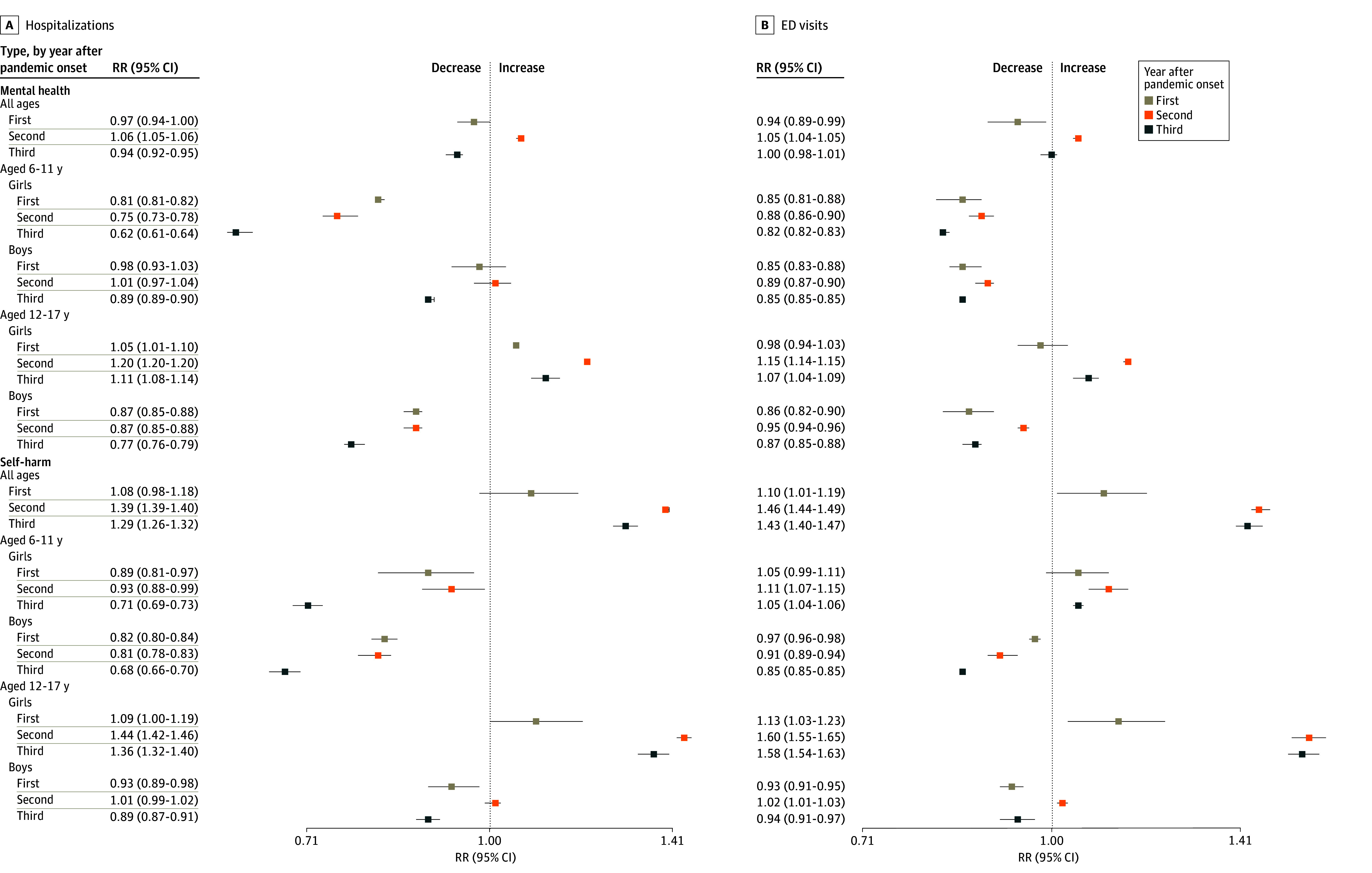
Rate Ratios (RRs) and 95% CIs for Hospitalizations and Emergency Department (ED) Visits Related to Mental Health and Self-Harm Among Children for 3 Years After the Pandemic Onset, Overall and by Age and Sex

In January 2016, rates of MH-related hospitalizations and ED visits were 54.9 (95% CI, 53.1-56.8) and 37.9 (95% CI, 35.9-39.9) per 100 000 children, respectively. In the prepandemic period, only rates of MH-related ED visits were rising, with an increase of 0.3% per month (95% CI, 0.1%-0.4%) (Figure 1A and B and Table 2). In the initial pandemic period, rates of MH-related hospitalizations and ED visits decreased by 48.5% (95% CI, −52.3% to −44.3%) and 48.4% (95% CI, −53.4% to −42.9%), respectively (Figure 1A and B and Table 2). In the first year after the pandemic onset, the rate of MH-related hospitalizations increased by 1.8% per month (95% CI, 1.0%-2.6%) and that of ED visits increased by 3.1% per month (95% CI, 1.9%-4.1%). Thereafter, all trends changed substantially, decreasing over the second and third years (Figure 1A and B and Table 2). Rates of MH-related hospitalizations and ED visits rose above expected rates only in the second year after the pandemic onset, with increases of 6.0% (RR, 1.06 [95% CI, 1.05-1.06]) for hospitalizations and 5.0% (RR, 1.05 [95% CI, 1.04-1.05]) for ED visits (Figure 2).

**Table 2.** Changes in Trends and Rates of Hospitalizations and ED Visits Related to Mental Health Conditions and Self-Harm Among Children, Using Log-Linear Regression Parameters

Group	COVID-19 pandemic period														
	Before onset (Jan 2016-Feb 2020)			Initial period (March-May 2020)		First year after onset (June 2020-May 2021)			Second year after onset (June 2021-May 2022)			Third year after onset (June 2022-May 2023)			
	Level, Jan 2016 (95% CI)^a^	Trend, % (95% CI)^b^	*P* value	Change in level, % (95% CI)^b^	*P* value	Change in trend, % (95% CI)^b^	*P* value	Trend, % (95% CI)^b^	Change in trend, % (95% CI)^b^	*P* value	Trend, % (95% CI)^b^	Change in trend, % (95% CI)^b^	*P* value	Trend, % (95% CI)^b^	Level, May 2023^a^
**All ages**															
MH-related hospitalizations	54.9 (53.1 to 56.8)	0.0 (−0.1 to 0.1)	.88	−48.5 (−52.3 to −44.3)	<.001	1.8 (1.0 to 2.6)	<.001	1.8 (1.0 to 2.6)	−2.3 (−3.6 to −1.1)	<.001	−0.6 (−1.2 to 0.0)	−0.4 (−1.6 to 0.9)	.55	−0.9 (−1.6 to −0.2)	48.5 (45.7 to 51.4)
MH-related ED visits	37.9 (35.9 to 39.9)	0.3 (0.1 to 0.4)	.004	−48.4 (−53.4 to −42.9)	<.001	2.9 (1.6 to 4.1)	<.001	3.1 (1.9 to 4.4)	−3.1 (−4.9 to −1.2)	.002	0.0 (−1.0 to 0.9)	−0.5 (−2.3 to 1.4)	.59	−0.5 (−1.6 to 0.5)	45.7 (41.9 to 49.9)
SH-related hospitalizations	9.9 (9.4 to 10.3)	0.2 (0.1 to 0.4)	.005	−37.1 (−42.7 to −30.9)	<.001	4.8 (3.6 to 5.9)	<.001	5.0 (3.9 to 6.1)	−4.4 (−6.0 to −2.7)	<.001	0.4 (−0.4 to 1.2)	−1.5 (−3.0 to 0.1)	.07	−1.1 (−2.0 to −0.1)	14.5 (13.4 to 15.7)
SH-related ED visits	6.5 (6.2 to 7.0)	0.7 (0.5 to 0.9)	<.001	−40.8 (−47.5 to −33.3)	<.001	4.3 (2.8 to 5.7)	<.001	5.0 (3.6 to 6.4)	−3.1 (−5.3 to −0.9)	.006	1.7 (0.6 to 2.8)	−2.2 (−4.2 to 0.1)	.04	−0.5 (−1.7 to 0.8)	16.3 (14.7 to 18.0)
**Children aged 6-11 y**															
Girls															
MH-related hospitalizations	12.5 (11.3 to 13.8)	0.4 (0.1 to 0.8)	.02	−53.5 (−59.9 to −46.1)	<.001	0.4 (−1.6 to 2.5)	.69	0.8 (−1.1 to 2.8)	−1.9 (−5.1 to 1.3)	.24	−1.1 (−2.8 to 0.6)	0.0 (−3.1 to 3.2)	.97	−1.2 (−2.9 to 0.6)	10.4 (8.9 to 12.0)
MH-related ED visits	11.6 (11.2 to 12.1)	0.3 (0.2 to 0.5)	<.001	−48.4 (−52.6 to −43.7)	<.001	2.0 (1.0 to 3.0)	<.001	2.3 (1.4 to 3.3)	−3.0 (−4.4 to −1.5)	<.001	−0.7 (−1.4 to 0.0)	0.9 (−0.5 to 2.4)	.20	0.2 (−0.6 to 1.1)	12.7 (11.9 to 13.6)
SH-related hospitalizations	0.6 (0.5 to 0.6)	1.1 (0.7 to 1.4)	<.001	−32.4 (−45.6 to −15.9)	<.001	4.7 (2.3 to 7.1)	<.001	5.8 (3.4 to 8.2)	−7.3 (−10.6 to-3.9)	<.001	−2.0 (−3.6 to −0.2)	1.5 (−1.9 to 5.1)	.37	−0.5 (−2.4 to 1.6)	1.0 (0.8 to 1.2)
SH-related ED visits	0.9 (0.8 to 0.9)	0.9 (0.5 to 1.2)	<.001	−37.3 (−49.1 to −22.8)	<.001	3.2 (0.9 to 5.5)	.006	4.1 (1.8 to 6.3)	−4.7 (−8.0 to −1.4)	.007	−0.9 (−2.5 to 0.8)	2.4 (−0.9 to 5.9)	.14	1.5 (−0.4 to 3.5)	2.0 (1.7 to 2.3)
Boys															
MH-related hospitalizations	37.7 (35.1 to 40.6)	−0.2 (−0.4 to 0.1)	.22	−61.0 (−66.3 to −54.8)	<.001	2.7 (1.0 to 4.5)	.002	2.6 (0.9 to 4.3)	−4.5 (−7.0 to −1.8)	.001	−2.0 (−3.3 to −0.7)	1.6 (−0.9 to 4.3)	.20	−0.4 (−1.8 to 1.1)	29.1 (25.7 to 32.8)
MH-related ED visits	13.5 (12.9 to 14.2)	0.2 (0.1 to 0.4)	.004	−48.4 (−53.6 to −42.5)	<.001	1.7 (0.5 to 2.9)	.006	1.9 (0.8 to 3.1)	−2.4 (−4.2 to −0.6)	.010	−0.5 (−1.4 to 0.3)	0.9 (−0.9 to 2.6)	.32	0.3 (−0.7 to 1.3)	14.4 (13.3 to 15.7)
SH-related hospitalizations	0.4 (0.4 to 0.5)	0.8 (0.2 to 1.3)	.005	−37.6 (−55.7 to −12.1)	.008	1.4 (−2.2 to 5.2)	.44	2.2 (−1.4 to 5.9)	−2.8 (−8.3 to 2.9)	.32	−0.7 (−3.4 to 2.0)	0.0 (−5.2 to 5.6)	.98	−0.7 (−3.8 to 2.5)	0.5 (0.4 to 0.7)
SH-related ED visits	0.9 (0.8 to 1.1)	1.1 (0.5 to 1.7)	<.001	−19.8 (−40.7 to 8.6)	.15	0.5 (−3.3 to 4.4)	.80	1.6 (−2.1 to 5.4)	−1.8 (−7.7 to 4.4)	.54	−0.3 (−3.2 to 2.8)	1.4 (−4.3 to 7.5)	.63	1.1 (−2.2 to 4.6)	2.1 (1.6 to 2.8)
**Adolescents aged 12-17 y**															
Girls															
MH-related hospitalizations	92.1 (87.0 to 97.6)	0.1 (−0.1 to 0.3)	.27	−32.8 (−37.7 to −27.5)	<.001	2.4 (1.2 to 3.5)	<.001	2.5 (1.4 to 3.6)	−2.3 (−4.0 to −0.5)	.01	0.2 (−0.8 to 1.1)	−1.3 (−2.9 to 0.4)	.14	−1.1 (−2.1 to −0.1)	105.0 (96.7 to 114.0)
MH-related ED visits	78.8 (75.2 to 82.5)	0.4 (0.2 to 0.5)	<.001	−56.2 (−60.3 to −51.6)	<.001	2.6 (1.5 to 3.7)	<.001	3.0 (1.9 to 4.1)	−2.3 (−3.9 to −0.6)	.009	0.6 (−0.2 to 1.5)	−1.5 (−3.1 to 0.1)	.06	−0.9 (−1.9 to 0.0)	108.6 (100.5 to 117.4)
SH-related hospitalizations	32.3 (31.3 to 33.5)	0.2 (0.1 to 0.3)	<.001	−44.5 (−48.5 to −40.2)	<.001	4.4 (3.5 to 5.2)	<.001	4.6 (3.8 to 5.4)	−3.4 (−4.7 to −2.2)	<.001	1.0 (0.4 to 1.6)	−2.3 (−3.5 to −1.1)	<.001	−1.3 (−2.0 to −0.6)	48.7 (45.9 to 51.6)
SH-related ED visits	19.6 (18.7 to 20.5)	0.6 (0.5 to 0.8)	<.001	−45.7 (−51.0 to −39.9)	<.001	4.6 (3.5 to 5.8)	<.001	5.3 (4.2 to 6.4)	−2.8 (−4.5 to −1.1)	.002	2.4 (1.5 to 3.2)	−3.3 (−4.8 to −1.7)	<.001	−1.0 (−1.9 to 0.0)	49.1 (45.4 to 53.2)
Boys															
MH-related hospitalizations	78.4 (76.7 to 80.2)	−0.2 (−0.3 to −0.2)	<.001	−50.9 (−53.3 to −48.4)	<.001	1.0 (0.4 to 1.5)	<.001	0.7 (0.2 to 1.2)	−1.7 (2.6 to −0.9)	<.001	−1.0 (−1.4 to −0.7)	−0.2 (−1.0 to 0.6)	.56	−1.3 (−1.7 to −0.8)	46.4 (44.6 to 48.2)
MH-related ED visits	48.0 (45.7 to 50.4)	0.1 (−0.1 to 0.3)	.19	−48.2 (−52.8 to −43.1)	<.001	2.5 (1.3 to 3.6)	<.001	2.6 (1.5 to 3.7)	−2.9 (−4.6 to −1.2)	.001	−0.4 (−1.3 to 0.5)	−0.5 (−2.2 to 1.2)	.54	−0.9 (−1.9 to 0.1)	43.4 (40.0 to 47.0)
SH-related hospitalizations	6.8 (6.3 to 7.4)	0.2 (0.0 to 0.5)	.09	−40.1 (−48.8 to −29.8)	<.001	2.4 (0.5 to 4.4)	.02	2.6 (0.8 to 4.6)	−3.1 (−6.0 to −0.1)	.04	−0.6 (−2.0 to 0.9)	−0.4 (−3.2 to 2.5)	.78	−1.0 (−2.6 to 0.7)	7.1 (6.2 to 8.1)
SH-related ED visits	5.1 (4.8 to 5.4)	1.0 (0.7 to 1.2)	<.001	−47.2 (−54.1 to −39.4)	<.001	1.2 (−0.2 to 2.8)	.10	2.2 (0.8 to 3.7)	−0.8 (−3.1 to 1.5)	.46	1.4 (0.2 to 2.5)	−1.9 (−4.1 to 0.2)	.08	−0.6 (−1.9 to 0.7)	10.2 (9.2 to 11.3)

Abbreviations: ED, emergency department; MH, mental health conditions; SH, self-harm.

^a^ Level corresponds to the monthly rate per 100 000 children estimated by the model.

^b^ Model estimates for trends and changes in level and trend are presented as percentage changes per month (95% CI).

Level and trend changes for MH-related hospitalizations and ED visits by age and sex are presented in Table 2 and illustrated in eFigures 1 and 2 in Supplement 1. After an initial decrease in rates of MH-related hospitalizations and ED visits in all groups and increasing trends in the first year following the pandemic onset, trends began decreasing in the second year for all groups except adolescent girls aged 12 to 17 years, in which trends decreased only in the third year following the pandemic onset (Table 2). In comparisons between estimated and expected MH-related hospitalization and ED visit rates, substantial increases were observed only for adolescent girls, essentially in the second year (20.0%; RR, 1.20 [95% CI, 1.20-1.20]; and 15.0%; RR, 1.15 [95% CI, 1.14-1.15], respectively) and third year (11.0%; RR, 1.11 [95% CI, 1.08-1.14]; and 7.0% [RR, 1.07 [95% CI, 1.04-1.09], respectively) after the pandemic onset.

**Figure 3.**
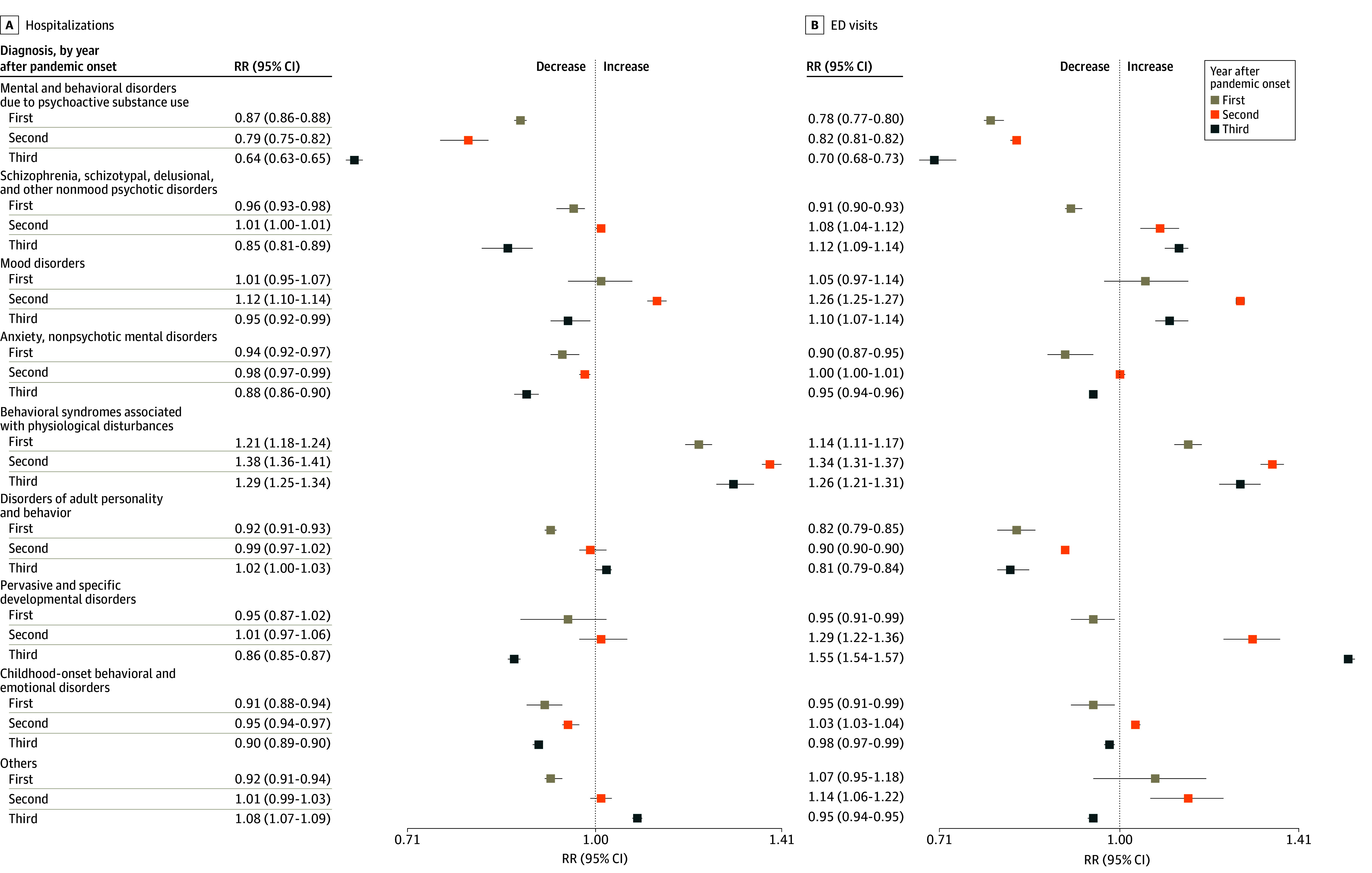
Rate Ratios (RRs) and 95% CIs for Hospitalizations and Emergency Department (ED) Visits Among Children for 3 Years After the Pandemic Onset, by Primary Mental Health Diagnosis Others includes mental disorders due to known physiological conditions, intellectual disabilities, unspecified mental disorders, and Z00-Z99 primary diagnoses associated with F00-F99 diagnostic codes (*International Statistical Classification of Diseases, Tenth Revision*) as secondary diagnoses.

Changes in medical diagnoses related to hospitalizations and ED visits for MH conditions are shown in Figure 3. Compared with expected rates, statistically significant increases were observed only in rates of hospitalizations related to the behavioral syndromes associated with physiological disturbances (mainly eating and sleep disorders) in all years after the pandemic onset (29.0% in the third year; RR, 1.29 [95% CI, 1.25-1.34]) and to mood disorders in the second year only (12.0%; RR, 1.12 [95% CI, 1.10-1.14]). In all years after the pandemic onset, increases in MH-related ED visit rates were observed for mood disorders (10.0%; RR, 1.10 [95% CI, 1.07-1.14] in the third year) and for behavioral syndromes associated with physiological disturbances (26.0%; RR, 1.26 [95% CI, 1.21-1.31] in the third year). In the second and third years after the pandemic onset, increases in MH-related ED visit rates were observed for schizophrenia or other psychotic disorders (12.0%; RR, 1.12 [95% CI, 1.09-1.14] in the third year) and pervasive or specific developmental disorders (55.0%; RR, 1.55 [95% CI, 1.54-1.57] in the third year).

### Hospitalizations and ED Visits Related to SH

Overall, we analyzed 108 245 hospitalizations and 87 162 ED visits related to SH. Compared with the prepandemic period, the proportion of girls increased for both SH-related hospitalizations (80.9% vs 85.3%) and ED visits (74.6% vs 79.4%), and that of patients with low socioeconomic status also increased for SH-related hospitalizations (19.0% vs 21.5%) (Table 1).

In January 2016, the estimated rates of SH-related hospitalizations and ED visits were 9.9 (95% CI, 9.4-10.3) and 6.5 (95% CI, 6.2-7.0) per 100 000 children, respectively. In the prepandemic period, rates of SH-related hospitalizations and ED visits were increased by 0.2% (95% CI, 0.1%-0.4%) and 0.7% (95% CI, 0.5%-0.9%) per month, respectively (Figure 1C and D and Table 2). In the initial pandemic period, rates of both SH-related hospitalizations and ED visits decreased statistically significantly (Figure 1C and D and Table 2). In the first year after the pandemic onset, the rate of hospitalizations increased by 5.0% per month (95% CI, 3.9%-6.1%) and that of ED visits increased by 5.0% per month (95% CI, 3.6%-6.4%). However, trend increases halted in the second year; in the third year, rates of SH-related hospitalizations and ED visits decreased by 1.1% (95% CI, −2.0% to −0.1%) and 0.5% (95% CI, −1.7% to 0.8%) per month, respectively. Compared with the expected rates, the rate of SH-related hospitalizations increased by 39.0% (RR, 1.39 [95% CI, 1.39-1.40]) in the second year and by 29.0% (RR, 1.29 [95% CI, 1.26-1.32]) in the third year after the pandemic onset. Increases in rates of SH-related ED visits were also substantial, with estimated increases of 46.0% (RR, 1.46 [95% CI, 1.44-1.49]) in the second year and 43.0% (RR, 1.43 [95% CI, 1.40-1.47]) in the third year following the pandemic (Figure 2).

Level and trend changes for SH-related hospitalizations and ED visits by age and sex are presented in Table 2 and illustrated in eFigures 3 and 4 in Supplement 1. After an initial decrease in rates of SH-related hospitalizations and ED visits in all groups and increasing trends in the first year following the pandemic onset, trends decreased in the second year for all groups except girls aged 12 to 17 years, for whom trends decreased only in the third year (Table 2). Compared with the expected rates of SH-related hospitalizations and ED visits, substantial increases were observed only for adolescent girls, essentially in the second year (44.0%; RR, 1.44 [95% CI, 1.42-1.46]; and 60.0%; RR, 1.60 [95% CI, 1.55-1.65], respectively) and third year (36.0%; RR, 1.36 [95% CI, 1.32-1.40]; and 58.0%; RR, 1.58 [95% CI, 1.54-1.63], respectively) after the pandemic onset (Figure 2). Increases were also observed among girls aged 6 to 11 years, but the overall rates were very low (Figure 2).

Details on all model estimates and validity are provided in eTable 1 and eFigure 5 in Supplement 1. All sensitivity analyses yielded similar estimates (eFigures 6 and 7 and eTables 2 and 3 in Supplement 1).

## Discussion

In this study, hospitalization and ED visit rates and trends related to MH conditions and intentional SH among children aged 6 to 17 years changed substantially after the onset of the COVID-19 pandemic. Overall, rates of MH-related hospitalizations and ED visits rose above expected rates only during the second year after the pandemic onset. However, rates of MH-related hospitalizations and ED visits among girls aged 12 to 17 years and among those diagnosed with behavioral syndromes associated with physiological disturbances, particularly eating and sleep disorders, persistently exceeded expected rates 3 years after the pandemic onset. Likewise, rates of SH-related hospitalizations and ED visits, specifically among girls aged 12 to 17 years, persistently exceeded expected rates after the pandemic onset.

Several studies worldwide have reported increases in ED visits and hospitalizations related to MH conditions and suicidality after the pandemic onset.^22,23,24,25,26,27^ Our temporal analysis uncovered a substantial reduction in both hospitalizations and ED visits in the initial pandemic period, potentially reflecting disrupted health care access and hesitancy to seek medical attention during the pandemic peak. Thereafter, a notable surge occurred in the first 2 years, especially for hospital resource use related to intentional SH, followed by a downward trend in the third year, suggesting a complex and evolving pattern. This finding may be interpreted as a gradual fading of the acute effects of the pandemic on MH, but it may also suggest a potential stabilization at a higher rate level, indicating a new baseline for MH care resource demands.^28^ In this context, real-time monitoring of MH and SH statistics is of major importance to inform future allocation of medical resources.

Furthermore, our findings highlight adverse outcomes of the pandemic and its consequences, revealing distinct disparities based on age and sex. Indeed, the rise in both MH- and SH-related hospitalizations and ED visits after the pandemic has been substantial and persistent among girls aged 12 to 17 years. Previous studies have already described the vulnerability of this specific group,^23,25,29,30,31^ but our study shows adverse outcomes persisting 3 years after the onset of the pandemic, notably with regard to SH acts. We also observed increases in SH-related ED visits among girls younger than 12 years, which was, however, not reflected in hospitalization rates. Future research should focus on the drivers of this sex disparity.

Rates of hospitalizations for behavioral syndromes associated with physiological disturbances (essentially eating and sleep disorders) increased substantially after the pandemic, along with rates of hospitalizations for mood disorders and SH. Because hospital resources are not indefinitely expandable, these severe patient cases exert increased strain on available resources, potentially limiting the capacity to care for individuals with other MH conditions and other patient populations. However, in the other age and sex groups in this study, we observed decreases in rates of both MH- and SH-related ED visits. This finding, which was described in a 2023 study,^29^ may potentially reflect a shift toward psychiatric care in outpatient or community-based settings,^32,33^ disparities in health care–seeking behavior, or MH issues that eventually manifest in ways not captured in the use of medical resources (eg, in violent encounters).^34^ This finding reinforces the importance of developing genuine public policies for the prevention of mental disorders, at all stages of life, and especially for children and adolescents. Particular attention must be paid to suicide prevention and its determinants. School-based MH programs and telemedicine services may offer viable avenues for delivering support to children struggling with MH challenges, including suicidal ideation.^35,36^

Social isolation resulting from school closures and cancellation of extracurricular activities due to lockdowns has been cited as the main trigger for depressive and anxiety symptoms in children.^7,37,38^ Yet unlike other countries, school closures in France were limited, and other additional stressors should be considered.^39^ The looming threat of new lockdowns and uncertainty about the future may have had a deep effect on mental well-being, contributing to a sense of helplessness and isolation.^3^ Economic hardships associated with the pandemic fueled parental stress and in-home conflicts, and resulted in radical changes in living conditions for many families.^40,41^ The cumulative burden of these stressors may have exacerbated preexisting MH conditions and triggered new ones, underlining the need to adopt a comprehensive approach that not only addresses immediate health care needs but also tackles the underlying psychosocial stressors.^42^

The COVID-19 pandemic accelerated the growth of social media. Although social media provide the opportunity for vital interactions during times of physical isolation, they may also contribute to peer pressure, body image dissatisfaction, and cyberbullying, particularly among adolescents.^43,44,45^ Constant exposure to pandemic-related news generated cyberchondria and added to the growing anxiety of young generations about their future amid climate change and environmental crisis.^42^ The findings of our study, in combination with those of the aforementioned studies, suggest that the complex interplay between social media use, MH outcomes, and sex should be further explored, and that promotion of healthy digital habits should be enhanced. Finally, a rise in domestic and community violence was observed during the pandemic.^46^ Witnessing or experiencing violent encounters is associated with depression, acute and posttraumatic stress, and insecurity.^46^ The amplified demand for MH services documented in our study may reasonably be thought to correlate with increased risk of violence among affected children and adolescents, especially girls, who are more prone to domestic violence.

### Limitations

Our study has some limitations. The RPU database does not cover the activity of all EDs in France, particularly that of psychiatric hospitals. Estimated monthly rates of MH- and SH-related ED visits may therefore be underestimated, but this did not affect our analysis of the trends and comparisons to expected rates. Also, the definition of the supplementary universal health coverage variable, used as a proxy for socioeconomic status in our study, was broadened in 2019. Therefore, the higher proportion of patients of low socioeconomic status in the postpandemic period may result from this change. Finally, changes in inpatient and acute care resources over time may affect the actual rate of hospitalizations and ED visits. However, we conducted a sensitivity analysis adjusting our model to potential variations in resources (total number of hospitalizations and ED visits), which yielded estimates consistent with our primary findings.

## Conclusions

This cross-sectional study found substantial changes in rates and trends of hospitalizations and ED visits related to MH conditions and intentional SH among children following the onset of the COVID-19 pandemic. Changes were more prominent and persistent overall among girls aged 12 to 17 years and for hospitalizations and ED visits related to SH. Future research should focus on identifying and addressing persistent stressors in children. Longer-term monitoring of the use of acute hospital resources related to MH should be pursued to guide allocation of health care resources and to tailor preventive interventions. Nevertheless, addressing the persisting deterioration of children’s well-being should involve a multifaceted approach encompassing not only health care initiatives but also educational and public health interventions.

## Notes

Supplement 1. **eMethods 1.** Evolution of the Annual Percentage of Coverage of Emergency Departments in France in the Activity of Emergency Departments Dataset (Résumé de Passage aux Urgences [RPU]) During the Study Period **eMethods 2.** Definition of Mental Health–Related Outcomes
eReferences
**eMethods 3.** Specific *ICD-10* Codes Associated With F00-F99 *ICD-10* Categories for Mental Health–Related Hospitalizations and Emergency Department (ED) Visits **eMethods 4.** Time-Series Log-Linear Regression Model **eTable 1.** Changes in the Trends and Rates of Mental Health (MH) and Self-Harm (SH) Hospitalizations and Emergency Department (ED) Visits: Log-Linear Regression Parameters **eTable 2.** Sensitivity Analyses for Monthly Rates of Hospitalizations and Emergency Department (ED) Visits Related to Mental Health Conditions **eTable 3.** Sensitivity Analyses for Monthly Rates of Hospitalizations and Emergency Department (ED) Visits Related to Self-Harm **eFigure 1.** Changes in Rates and Trends of Mental Health Hospitalizations and ED Visits in Girls and Boys Aged 6 to 11 Years in France **eFigure 2.** Changes in Rates and Trends of Mental Health Hospitalizations and ED Visits in Girls and Boys Aged 12 to 17 Years in France **eFigure 3.** Changes in Rates and Trends of Self-Harm–Related Hospitalizations and ED Visits in Girls and Boys Aged 6 to 11 Years in France **eFigure 4.** Changes in Rates and Trends of Self-Harm–Related Hospitalizations and ED Visits in Girls and Boys Aged 12 to 17 Years in France **eFigure 5.** Correlograms and Residual Analysis of Primary Analyses **eFigure 6.** Correlograms and Residual Analysis of Sensitivity Analyses: Mental Health **eFigure 7.** Correlograms and Residual Analysis of Sensitivity Analyses: Self-Harm

Supplement 2.
Data Sharing Statement
