# Supplementary material for: Pediatric Hospitalizations and Emergency Department Visits Related to Mental Health Conditions and Self-Harm
Source: JAMA Netw Open. 2024 Oct 29;7(10):e2441874. doi: 10.1001/jamanetworkopen.2024.41874 (PMC11522941; doi:10.1001/jamanetworkopen.2024.41874)
Supplement: Supplement 1. — eMethods 1. Evolution of the Annual Percentage of Coverage of Emergency Departments in France in the Activity of Emergency Departments Dataset (Résumé de Passage aux Urgences [RPU]) During the Study Period eMethods 2. Definition of Mental Health–Related Outcomes eReferences eMethods 3. Specific ICD-10 Codes Associated With F00-F99 ICD-10 Categories for Mental Health–Related Hospitalizations and Emergency Department (ED) Visits eMethods 4. Time-Series Log-Linear Regression Model eTable 1. Changes in the Trends and Rates of Mental Health (MH) and Self-Harm (SH) Hospitalizations and Emergency Department (ED) Visits: Log-Linear Regression Parameters eTable 2. Sensitivity Analyses for Monthly Rates of Hospitalizations and Emergency Department (ED) Visits Related to Mental Health Conditions eTable 3. Sensitivity Analyses for Monthly Rates of Hospitalizations and Emergency Department (ED) Visits Related to Self-Harm eFigure 1. Changes in Rates and Trends of Mental Health Hospitalizations and ED Visits in Girls and Boys Aged 6 to 11 Years in France eFigure 2. Changes in Rates and Trends of Mental Health Hospitalizations and ED Visits in Girls and Boys Aged 12 to 17 Years in France eFigure 3. Changes in Rates and Trends of Self-Harm–Related Hospitalizations and ED Visits in Girls and Boys Aged 6 to 11 Years in France eFigure 4. Changes in Rates and Trends of Self-Harm–Related Hospitalizations and ED Visits in Girls and Boys Aged 12 to 17 Years in France eFigure 5. Correlograms and Residual Analysis of Primary Analyses eFigure 6. Correlograms and Residual Analysis of Sensitivity Analyses: Mental Health eFigure 7. Correlograms and Residual Analysis of Sensitivity Analyses: Self-Harm [file jamanetwopen-e2441874-s001.pdf]

Supplementary Online Content

Valtuille Z, Trebossen V, Ouldali N, et al. Pediatric hospitalizations and emergency department visits related to mental health conditions and self-harm. *JAMA Netw Open*. 2024;7(10):e2441874. doi:10.1001/jamanetworkopen.2024.41874

eMethods 1. Evolution of the Annual Percentage of Coverage of Emergency Departments in France in the Activity of Emergency Departments Dataset (Résumé de Passage aux Urgences [RPU]) During the Study Period

eMethods 2. Definition of Mental Health–Related Outcomes

eReferences

eMethods 3. Specific *ICD-10* Codes Associated With F00-F99 *ICD-10* Categories for Mental Health–Related Hospitalizations and Emergency Department (ED) Visits

eMethods 4. Time-Series Log-Linear Regression Model

eTable 1. Changes in the Trends and Rates of Mental Health (MH) and Self-Harm (SH) Hospitalizations and Emergency Department (ED) Visits: Log-Linear Regression Parameters

eTable 2. Sensitivity Analyses for Monthly Rates of Hospitalizations and Emergency Department (ED) Visits Related to Mental Health Conditions

eTable 3. Sensitivity Analyses for Monthly Rates of Hospitalizations and Emergency Department (ED) Visits Related to Self-Harm

eFigure 1. Changes in Rates and Trends of Mental Health Hospitalizations and ED Visits in Girls and Boys Aged 6 to 11 Years in France

eFigure 2. Changes in Rates and Trends of Mental Health Hospitalizations and ED Visits in Girls and Boys Aged 12 to 17 Years in France

eFigure 3. Changes in Rates and Trends of Self-Harm–Related Hospitalizations and ED Visits in Girls and Boys Aged 6 to 11 Years in France

eFigure 4. Changes in Rates and Trends of Self-Harm–Related Hospitalizations and ED Visits in Girls and Boys Aged 12 to 17 Years in France

eFigure 5. Correlograms and Residual Analysis of Primary Analyses

eFigure 6. Correlograms and Residual Analysis of Sensitivity Analyses: Mental Health

eFigure 7. Correlograms and Residual Analysis of Sensitivity Analyses: Self-Harm

This supplementary material has been provided by the authors to give readers additional information about their work.

© 2024 Valtuille Z et al. *JAMA Network Open.*

eMethods 1. Evolution of the Annual Percentage of Coverage of Emergency Departments in France in the Activity of Emergency Departments Dataset (Résumé de Passage aux Urgences [RPU]) During the Study Period

Year	Annual percentage of coverage
2016	84%
2017	90%
2018	93%
2019	92%
2020	92%
2021	95%
2022	95%
2023	95%

© 2024 Valtuille Z et al. *JAMA Network Open.*

eMethods 2. Definition of Mental Health–Related Outcomes

In the MCO and RIM-P datasets of the PMSI database, medical diagnoses are derived from the anonymous discharge summaries (*Résumés de Sortie Anonyme*, RSA) established at the end of each hospital stay and are classified as follows:

- Principal diagnoses (DP): medical reason justifying the hospitalization.
- Related diagnoses (DR): diagnosis specifying the DP
- Associated diagnoses (DA): morbidity associated to DP

One RSA contains one DP and as many DR or DA as needed to describe the patient's medical condition through the hospital stay. In the manuscript, DP and DR are referred to as 'principal diagnoses' and DA as 'secondary diagnoses' (1).

- Hospitalizations related to mental health conditions were defined as overnight hospital stays with at least: 1) one principal diagnosis (DP or DR) coded using ICD-10 F00-F99 (mental and behavioral disorders) codes or 2) one principal diagnosis (DP) coded using ICD-10 Z00-Z99 (factors influencing health status and contact with health services) codes and associated diagnosis (DA) using ICD-10 F00-F99 codes.
- Hospitalizations related to self-harm were defined as hospital stays with a DA coded using ICD-10 X60-X84 (intentional self-harm) codes. (2-4). This includes hospital stays with F00-F99 ICD-10 codes as a principal diagnosis (DP) and associated diagnosis DA coded with X60-X84 ICD-10 codes.

In the RPU dataset of the PMSI database, medical diagnoses are derived from the anonymous discharge summaries (*Résumés de Sortie Anonyme*, RSA) established at the end of each emergency department visit and are classified as follows:

- Principal diagnoses (DP): medical reason justifying the ED visit.
- Associated diagnoses (DA): morbidity associated to DP

One RSA contains one DP and as many DAs as needed to describe the patient's medical condition through the visit. In the manuscript, DP is referred to as 'principal diagnosis' and DA as 'secondary diagnoses'.

- Emergency department visits related to mental-health conditions were identified as visits with at least one diagnosis (DP or DA) coded using F00-F99 (mental and behavioral disorders) ICD-10 codes.

- Emergency department visits related to self-harm, were identified as visits with at least one diagnosis (DP or DA) coded using X60-X84 (intentional self-harm). Because coding using the X60-X84 ICD10 codes may be incomplete in the RPU dataset, we also included in the analysis, emergency department visits with at least one diagnosis (DP or DA) coded using the following codes as recommended in previous studies using RPU data (2, 3): T39 (poisoning by nonopioid analgesics, antipyretics and antirheumatics); T42-T43 (intoxication through psychotropics); T50 (poisoning by diuretics and other and unspecified drugs, medications and biological substances); T60 (toxic effect of pesticides); T71 (asphyxiation); Y870 (sequelae of intentional self-harm, assault and events of undetermined intent); R458 (suicidal ideation) ICD-10 codes.

Mental health hospitalizations and emergency department visits were classified as follows based on ICD-10 codes for the principal diagnosis:

1. Mental and behavioral disorders due to psychoactive substance use: F10-F19
2. Schizophrenia, schizotypal, delusional, and other non-mood psychotic disorders: F20-F29
3. Mood disorders: F30-F39
4. Anxiety, dissociative, stress-related, somatoform and other nonpsychotic mental disorders: F40-F49
5. Behavioral syndromes associated with physiological disturbances and physical factors: F50-F59
6. Disorders of adult personality and behavior: F60-F69
7. Pervasive and specific developmental disorders: F80-F89
8. Childhood onset behavioral and emotional disorders: F90-F98
9. Others: F00-F09 (mental disorders due to known physiological conditions), F70-F79 (intellectual disabilities), F99 (unspecified mental disorder), and Z00-Z99 associated with F00-F99 codes as secondary diagnoses

Self-harm hospitalizations were further classified based on the ICD-10 classification as follows:

1. Drug self-poisoning: X60-X64
2. Self-poisoning by other products (alcohol, solvents, gases, pesticides, products chemicals): X65-X69
3. Violent means: X70-X82
4. Other means: X83-X84

© 2024 Valtuille Z et al. *JAMA Network Open.*

Self-harm emergency department visits were further classified based on the ICD-10 classification as follows:

1. Drug self-poisoning: X60-X64 + T39 + T42-T43 + T50 codes
2. Self-poisoning by other products (alcohol, solvents, gases, pesticides, products chemicals): X65-X69 +T60 codes
3. Violent means: X70-X82 + T71 codes
4. Other means: X83-X84
5. Sequelae of intentional self-harm, assault and events of undetermined intent: Y870
6. Suicidal ideation: R458

For the sensitivity analysis, we used a more stringent definition of hospital stays or emergency department visits related to intentional self-harm and suicide attempts in the PMSI database. Indeed, principal analysis was performed using the definition of intentional self-harm implemented in several previous publications (4,5) but in the sensitivity analysis, we applied the definition recommended by the PMSI coding system which defines intentional self-harm hospitalizations or emergency department visits as those with a DP coded using ICD-10 S00-T98 (Injury, poisoning and certain other consequences of external causes) codes and a DA coded using ICD-10 X60-X84 (Intentional self-harm) code (6).

eReferences

1)https://www.atih.sante.fr/sites/default/files/public/content/1856/GUIDE_METHODO_MCO_2012_vs_2011_2_1.pdf (accessed May 2024)
2) https://www.santepubliquefrance.fr/content/download/182527/2307205 (accessed May 2024)
3) Pirard Philippe, Chin Francis, Khiréddine Imane, Tebeka Sarah, Regnault Nolwenn. Bulletin épidémiologique hebdomadaire, 2023, n°. 12-13, p. 230-237 https://www.santepubliquefrance.fr/maladies-et-traumatismes/sante-mentale/suicides-et-tentatives-de-suicide/documents/article/hospitalisations-pour-tentative-de-suicide-dans-les-etablissements-de-soins-aigus-en-france-lors-de-l-infection-a-la-covid-19-tendances-temporell (accessed May 2024)
4) Jollant, F., et al. (2021). "Hospitalization for self-harm during the early months of the COVID-19 pandemic in France: A nationwide retrospective observational cohort study." Lancet Reg Health Eur **6**: 100102.
5) Site internet de Santé publique France, dossier thématique Suicides et tentatives de suicide https://www.santepubliquefrance.fr/maladies-et-traumatismes/sante-mentale/suicides-et-tentatives-de-suicide (accessed May 2024)
6) Épidémiologie des tentatives de suicide en région Centre - Données PMSI 2004-2006. https://www.google.com/url?sa=t&rct=j&q=&esrc=s&source=web&cd=&cad=rja&uact=8&ved=2ahUKEwi5kMTJy6KAAxUqxgIHHdTdABIQFnoECBMQAQ&url=https%3A%2F%2Fwww.esante-centre.fr%2Fportail_pro%2Fminisite_25%2Fmedia-files%2F56389%2Fplaquette-2004-2006.pdf&usg=AOvVaw2zRC1ORap12vPIp-m65XtU&opi=89978449 (accessed May 2024)

eMethods 3. Specific *ICD-10* Codes Associated With F00-F99 *ICD-10* Categories for Mental Health–Related Hospitalizations and Emergency Department (ED) Visits

**ICD-10**<br>**F00-F99 Mental, Behavioral and Neurodevelopmental disorders**	**Hospitalizations**	**ED visits**
**F00-F09 Mental disorders due to known physiological conditions**		
F01 Vascular dementia	3	23
F02 Dementia in other diseases classified elsewhere	5	31
F03 Unspecified dementia	11	43
F04 Organic amnesic syndrome, not induced by alcohol and other psychoactive substances	12	29
F05 Delirium, not induced by alcohol and other psychoactive substances	124	75
F06 Other mental disorders due to brain damage and dysfunction and to physical disease	1,498	1,818
F07 Personality and behavioral disorders due to brain disease, damage and dysfunction	1,029	6,379
F09 Unspecified organic or symptomatic mental disorder	49	231
**F10-F19 Mental and behavioral disorders due to psychoactive substance use**		
F10 Mental and behavioral disorders due to use of alcohol	9,970	26,335
F11 Mental and behavioral disorders due to use of opioids	352	782
F12 Mental and behavioral disorders due to use of cannabinoids	3,068	2,858
F13 Mental and behavioral disorders due to use of sedatives or hypnotics	346	1,212
F14 Mental and behavioral disorders due to use of cocaine	155	161
F15 Mental and behavioral disorders due to use of other stimulants, including caffeine	125	212
F16 Mental and behavioral disorders due to use of hallucinogens	102	167
F17 Mental and behavioral disorders due to use of tobacco	618	77
F18 Mental and behavioral disorders due to use of volatile solvents	28	147
F19 Mental and behavioral disorders due to multiple drug use and use of other psychoactive substances	921	1,444
**F20-F29 Schizophrenia, schizotypal, delusional, and other non-mood psychotic disorders**		
F20 Schizophrenia	6,870	967
F21 Schizotypal disorder	1,251	36
F22 Persistent delusional disorders	2,433	1,177
F23 Acute and transient psychotic disorders	5,405	2,999
F24 Induced delusional disorder	61	15
F25 Schizoaffective disorders	1,527	111
F28 Other nonorganic psychotic disorders	1,740	700
F29 Unspecified nonorganic psychosis	3,005	2,283
**F30-F39 Mood disorders**		
F30 Manic episode	724	238
F31 Bipolar affective disorder	2,640	907
F32 Depressive episode	62,942	54,541
F33 Recurrent depressive disorder	4,638	2,562
F34 Persistent mood (affective) disorders	1,855	2,147
F38 Other mood (affective) disorders	1,241	1,135
F39 Unspecified mood (affective) disorder	1,853	4,943
**F40-F49 Anxiety, dissociative, stress-related, somatoform and other nonpsychotic mental disorders**		
F40 Phobic anxiety disorders	6,464	2,429
F41 Other anxiety disorders	39,188	125,150
F42 Obsessive-compulsive disorder	2,837	648
F43 Reaction to severe stress, and adjustment disorders	38,431	13,596
F44 Dissociative (conversion) disorders	2,676	2,411
F45 Somatoform disorders	6,241	16,328
F48 Other neurotic disorders	1,149	832

© 2024 Valtuille Z et al. *JAMA Network Open.*

**F50-F59 Behavioral syndromes associated with physiological disturbances and physical factors**		
F50 Eating disorders	27,185	8,928
F51 Nonorganic sleep disorders	3,038	1,283
F52 Sexual dysfunction, not caused by organic disorder or disease	33	56
F53 Mental and behavioral disorders associated with the puerperium, not elsewhere classified	66	133
F54 Psychological and behavioral factors associated with disorders or diseases classified elsewhere	397	412
F55 Abuse of non-dependence-producing substances	54	88
F59 Unspecified behavioral syndromes associated with physiological disturbances and physical factors	121	184
**F60-F69 Disorders of adult personality and behavior**		
F60 Specific personality disorders	17,155	6,770
F61 Mixed and other personality disorders	334	65
F62 Enduring personality changes, not attributable to brain damage and disease	116	84
F63 Habit and impulse disorders	970	134
F64 Gender identity disorders	489	38
F65 Disorders of sexual preference	29	24
F66 Psychological and behavioral disorders associated with sexual development and orientation	96	96
F68 Other disorders of adult personality and behavior	597	665
F69 Unspecified disorder of adult personality and behavior	443	3,150
**F70-F79 Intellectual disabilities**		
F70 Mild mental retardation	3,359	139
F71 Moderate mental retardation	2,441	84
F72 Severe mental retardation	1,433	43
F73 Profound mental retardation	1,264	17
F78 Other mental retardation	278	48
F79 Unspecified mental retardation	1,558	127
**F80-F89 Pervasive and specific developmental disorders**		
F80 Specific developmental disorders of speech and language	2,787	36
F81 Specific developmental disorders of scholastic skills	2,999	49
F82 Specific developmental disorder of motor function	755	19
F83 Mixed specific developmental disorders	3,912	27
F84 Pervasive developmental disorders	53,933	1,285
F88 Other disorders of psychological development	2,852	460
F89 Unspecified disorder of psychological development	1,745	724
**F90-F98 Childhood onset behavioral and emotional disorders**		
F90 Hyperkinetic disorders	12,462	323
F91 Conduct disorders	39,933	13,627
F92 Mixed disorders of conduct and emotions	31,513	7,686
F93 Emotional disorders with onset specific to childhood	15,733	6,967
F94 Disorders of social functioning with onset specific to childhood and adolescence	13,514	959
F95 Tic disorders	744	600
F98 Other behavioral and emotional disorders with onset usually occurring in childhood and adolescence	18,560	12,786
**F99 Mental disorder, not otherwise specified**	1,761	985

© 2024 Valtuille Z et al. *JAMA Network Open.*

eMethods 4. Time-Series Log-Linear Regression Model

The log-linear regression model can be written as:

Log(Yt) = $\beta_0 + \beta_1$ * time$_t$ + β_2 *initial pandemic period+ β_3 * remaining level after initial pandemic period + β_4 * time after end of the initial pandemic period + β_5 * time after end of the first post-pandemic period + β_6 * time after end of the second post-pandemic period + ϵ_t

Yt: monthly rate (per 100,000 children) of mental health and self-harm hospitalizations and emergency department (ED) visits in metropolitan France. Yt is in a logarithmic base, therefore model's estimates need to be exponentiated to be interpreted as mean percentage changes.

exp(β_0): estimate of the baseline level (rate per 100,000 children and adolescents) in January 1, 2016

exp(β_1): estimate of the change in the level per month (trend) in the pre-pandemic period.

Time$_t$: time elapsed since the beginning of the study; continuous variable measured in months ranging from January 1, 2016 to May 31, 2023.

exp(β_2): estimate of the change in level at the initial pandemic period.

Initial pandemic period: a categorical variable coded 0 before February 29, 2020 coded 1 from March 1 to May 31, 2020 and coded 0 again after Jun 1, 2020.

exp(β_3): estimate of the remaining change in level after the initial pandemic period.

Remaining level after initial pandemic period: a categorical variable coded 0 before May 31, 2020 and coded 1 after Jun 1, 2020.

exp(β_4): estimate of the change in trend after the beginning of the first post-pandemic year

Time after end of the initial pandemic period; continuous variable counting the number of months from June 1, 2020 to May 31, 2023.

exp($\beta_1 + \beta_4$): estimate of the new trend in the first post-pandemic year

exp(β_5): estimate of the change in trend after the beginning of the second post-pandemic year

Time after the end of the first post-pandemic year; continuous variable counting the number of months from June 1, 2021 to May 31, 2023.

exp($\beta_1 + \beta_4 + \beta_5$): estimate of the new trend in the second post-pandemic year

exp(β_6): estimate of the change in trend after the beginning of the third post-pandemic year

© 2024 Valtuille Z et al. *JAMA Network Open.*

Time after the end of the second post-pandemic year; continuous variable counting the number of months from June 1, 2022 to May 31, 2023.

$\exp(\beta_1 + \beta_4 + \beta_5 + \beta_6)$: estimate of the new trend in the third post-pandemic year

ϵ_t : the model's residual error

© 2024 Valtuille Z et al. *JAMA Network Open.*

eTable 1. Changes in the Trends and Rates of Mental Health (MH) and Self-Harm (SH) Hospitalizations and Emergency Department (ED) Visits: Log-Linear Regression Parameters

This table comprises the coefficients as calculated by the log-linear regression.

Model	Before onset period (Jan 2016 – Feb 2020)			Initial pandemic period (March – May 2020)		First year after pandemic onset (June 2020 – May 2021)					Second year after pandemic onset (June 2021 – May 2022)			Third year after pandemic onset (June 2022 – May 2023)			
	Level Jan. 2016 (95%CI)[a]	Trend (95%CI)	p-value	Change in level (95%CI)	p-value	Change in level (95%CI)	p-value	Change in trend (95%CI)	p-value	Trend (95%CI)	Change in trend (95%CI)	p-value	Trend (95%CI)	Change in trend (95%CI)	p-value	Trend (95%CI)	Level May 2023[a]
**All ages**																	
MH hospitalizations	54.913 (53.078 to 56.811)	1.000 (0.999 to 1.001)	*0.88*	0.515 (0.477 to 0.557)	*<0.001*	0.862 (0.797 to 0.931)	*<0.001*	1.018 (1.010 to 1.026)	*<0.001*	1.018 (1.01 to 1.026)	0.977 (0.964 to 0.989)	*<0.001*	0.994 (0.988 to 1.000)	0.996 (0.984 to 1.009)	*0.56*	0.991 (0.984 to 0.998)	48.5 (45.7 to 51.4)
MH ED visits	37.881 (35.922 to 39.946)	1.003 (1.001 to 1.004)	*0.005*	0.516 (0.466 to 0.571)	*<0.001*	0.777 (0.694 to 0.871)	*<0.001*	1.029 (1.016 to 1.041)	*<0.001*	1.031 (1.019 to 1.044)	0.969 (0.951 to 0.988)	*0.002*	1.0 (0.990 to 1.009)	0.995 (0.977 to 1.014)	*0.59*	0.995 (0.984 to 1.005)	45.7 (41.9 to 49.9)
SH hospitalizations	9.882 (9.436 to 10.349)	1.002 (1.001 to 1.004)	*0.005*	0.629 (0.573 to 0.691)	*<0.001*	0.788 (0.712 to 0.872)	*<0.001*	1.048 (1.036 to 1.059)	*<0.001*	1.05 (1.039 to 1.061)	0.956 (0.940 to 0.973)	*<0.001*	1.004 (0.996 to 1.012)	0.985 (0.970 to 1.001)	*0.07*	0.989 (0.98 to 0.999)	14.5 (13.4 to 15.7)
SH ED visits	6.541 (6.152 to 6.954)	1.007 (1.005 to 1.009)	*<0.001*	0.592 (0.525 to 0.667)	*<0.001*	0.830 (0.727 to 0.948)	*0.007*	1.043 (1.028 to 1.057)	*<0.001*	1.05 (1.036 to 1.064)	0.969 (0.947 to 0.991)	*0.006*	1.017 (1.006 to 1.028)	0.978 (0.958 to 0.999)	*0.04*	0.995 (0.983 to 1.008)	16.3 (14.7 to 18.0)
**Girls: 6-11 years**																	
MH hospitalizations	12.482 (11.292 to 13.796)	1.004 (1.001 to 1.008)	*0.02*	0.465 (0.401 to 0.539)	*<0.001*	0.793 (0.657 to 0.957)	*0.02*	1.004 (0.984 to 1.025)	*0.69*	1.008 (0.989 to 1.028)	0.981 (0.949 to 1.013)	*0.24*	0.989 (0.972 to 1.006)	1.000 (0.969 to 1.032)	*0.98*	0.988 (0.971 to 1.006)	10.4 (8.9 to 12.0)
MH ED visits	11.612 (11.154 to 12.089)	1.003 (1.002 to 1.005)	*<0.001*	0.516 (0.474 to 0.563)	*<0.001*	0.742 (0.678 to 0.812)	*<0.001*	1.020 (1.010 to 1.030)	*<0.001*	1.023 (1.014 to 1.033)	0.970 (0.956 to 0.985)	*<0.001*	0.993 (0.986 to 1.0)	1.009 (0.995 to 1.024)	*0.2*	1.002 (0.994 to 1.011)	12.7 (11.9 to 13.6)
SH hospitalizations	0.583 (0.530 to 0.642)	1.011 (1.007 to 1.014)	*<0.001*	0.676 (0.544 to 0.841)	*<0.001*	0.654 (0.526 to 0.815)	*<0.001*	1.047 (1.023 to 1.071)	*<0.001*	1.058 (1.034 to 1.082)	0.927 (0.894 to 0.961)	*<0.001*	0.98 (0.964 to 0.998)	1.015 (0.981 to 1.051)	*0.38*	0.995 (0.976 to 1.016)	1.0 (0.8 to 1.2)

© 2024 Valtuille Z et al. *JAMA Network Open.*

SH ED visits	0.854 (0.779 to 0.937)	1.009 (1.005 to 1.012)	*<0.001*	0.627 (0.509 to 0.772)	*<0.001*	0.852 (0.691 to 1.051)	*0.133*	1.032 (1.009 to 1.055)	*0.006*	1.041 (1.018 to 1.063)	0.953 (0.920 to 0.986)	*0.007*	0.991 (0.975 to 1.008)	1.024 (0.991 to 1.059)	*0.15*	1.015 (0.996 to 1.035)	2.0 (1.7 to 2.3)
**Boys: 6-11 years**																	
MH hospitalizations	37.736 (35.070 to 40.604)	0.998 (0.996 to 1.001)	*0.22*	0.390 (0.337 to 0.452)	*<0.001*	0.818 (0.697 to 0.960)	*0.014*	1.027 (1.010 to 1.045)	*0.002*	1.026 (1.009 to 1.043)	0.955 (0.930 to 0.982)	*0.001*	0.98 (0.967 to 0.993)	1.016 (0.991 to 1.043)	*0.21*	0.996 (0.982 to 1.011)	29.1 (25.7 to 32.8)
MH ED visits	13.507 (12.861 to 14.185)	1.002 (1.001 to 1.004)	*0.004*	0.516 (0.464 to 0.575)	*<0.001*	0.765 (0.685 to 0.854)	*<0.001*	1.017 (1.005 to 1.029)	*0.006*	1.019 (1.008 to 1.031)	0.976 (0.958 to 0.994)	*0.01*	0.995 (0.986 to 1.003)	1.009 (0.991 to 1.026)	*0.33*	1.003 (0.993 to 1.013)	14.4 (13.3 to 15.7)
SH hospitalizations	0.440 (0.378 to 0.512)	1.008 (1.002 to 1.013)	*0.005*	0.624 (0.443 to 0.879)	*0.008*	0.748 (0.530 to 1.058)	*0.1*	1.014 (0.978 to 1.052)	*0.45*	1.022 (0.986 to 1.059)	0.972 (0.917 to 1.029)	*0.32*	0.993 (0.966 to 1.02)	1.000 (0.948 to 1.056)	*0.99*	0.993 (0.962 to 1.025)	0.5 (0.4 to 0.7)
SH ED visits	0.922 (0.776 to 1.096)	1.011 (1.005 to 1.017)	*<0.001*	0.802 (0.593 to 1.086)	*0.151*	0.941 (0.659 to 1.344)	*0.74*	1.005 (0.967 to 1.044)	*0.803*	1.016 (0.979 to 1.054)	0.982 (0.923 to 1.044)	*0.55*	0.997 (0.968 to 1.028)	1.014 (0.957 to 1.075)	*0.64*	1.011 (0.978 to 1.046)	2.1 (1.6 to 2.8)
**Girls: 12-17 years**																	
MH hospitalizations	92.149 (87.002 to 97.601)	1.001 (0.999 to 1.003)	*0.28*	0.672 (0.623 to 0.725)	*<0.001*	0.902 (0.817 to 0.997)	*0.04*	1.024 (1.012 to 1.035)	*<0.001*	1.025 (1.014 to 1.036)	0.977 (0.960 to 0.995)	*0.01*	1.002 (0.992 to 1.011)	0.987 (0.971 to 1.004)	*0.14*	0.989 (0.979 to 0.999)	105.0 (96.7 to 114.0)
MH ED visits	78.758 (75.228 to 82.453)	1.004 (1.002 to 1.005)	*<0.001*	0.438 (0.397 to 0.484)	*<0.001*	0.831 (0.750 to 0.922)	*<0.001*	1.026 (1.015 to 1.037)	*<0.001*	1.03 (1.019 to 1.041)	0.977 (0.961 to 0.994)	*0.009*	1.006 (0.998 to 1.015)	0.985 (0.969 to 1.001)	*0.06*	0.991 (0.981 to 1.0)	108.6 (100.5 to 117.4)
SH hospitalizations	32.336 (31.258 to 33.451)	1.002 (1.001 to 1.003)	*<0.001*	0.555 (0.515 to 0.598)	*<0.001*	0.820 (0.759 to 0.885)	*<0.001*	1.044 (1.035 to 1.052)	*<0.001*	1.046 (1.038 to 1.054)	0.966 (0.953 to 0.978)	*<0.001*	1.01 (1.004 to 1.016)	0.977 (0.965 to 0.989)	*<0.001*	0.987 (0.98 to 0.994)	48.7 (45.9 to 51.6)
SH ED visits	19.572 (18.681 to 20.505)	1.006 (1.005 to 1.008)	*<0.001*	0.543 (0.490 to 0.601)	*<0.001*	0.829 (0.746 to 0.921)	*<0.001*	1.046 (1.035 to 1.058)	*<0.001*	1.053 (1.042 to 1.064)	0.972 (0.955 to 0.989)	*0.002*	1.024 (1.015 to 1.032)	0.967 (0.952 to 0.983)	*<0.001*	0.99 (0.981 to 1.000)	49.1 (45.4 to 53.2)
**Boys: 12-17 years**																	
MH hospitalizations	78.409 (76.692 to 80.164)	0.998 (0.997 to 0.998)	*<0.001*	0.491 (0.467 to 0.516)	*<0.001*	0.814 (0.774 to 0.857)	*<0.001*	1.010 (1.004 to 1.015)	*<0.001*	1.007 (1.002 to 1.012)	0.983 (0.974 to 0.991)	*<0.001*	0.99 (0.986 to 0.993)	0.998 (0.990 to 1.006)	*0.57*	0.987 (0.983 to 0.992)	46.4 (44.6 to 48.2)

© 2024 Valtuille Z et al. *JAMA Network Open.*

MH ED visits	48.036 (45.747 to 50.439)	1.001 (0.999 to 1.003)	*0.19*	0.518 (0.472 to 0.569)	*<0.001*	0.731 (0.659 to 0.812)	*<0.001*	1.025 (1.013 to 1.036)	*<0.001*	1.026 (1.015 to 1.037)	0.971 (0.954 to 0.988)	*0.001*	0.996 (0.987 to 1.005)	0.995 (0.978 to 1.012)	*0.54*	0.991 (0.981 to 1.001)	43.4 (40.0 to 47.0)
SH hospitalizations	6.849 (6.303 to 7.443)	1.002 (1.000 to 1.005)	*0.09*	0.599 (0.512 to 0.702)	*<0.001*	0.798 (0.668 to 0.953)	*0.01*	1.024 (1.005 to 1.044)	*0.02*	1.026 (1.008 to 1.046)	0.969 (0.940 to 0.999)	*0.04*	0.994 (0.98 to 1.009)	0.996 (0.968 to 1.025)	*0.78*	0.99 (0.974 to 1.007)	7.1 (6.2 to 8.1)
SH ED visits	5.056 (4.755 to 5.376)	1.010 (1.007 to 1.012)	*<0.001*	0.528 (0.459 to 0.606)	*<0.001*	0.857 (0.745 to 0.985)	*0.03*	1.012 (0.998 to 1.028)	*0.10*	1.022 (1.008 to 1.037)	0.992 (0.969 to 1.015)	*0.47*	1.014 (1.002 to 1.025)	0.981 (0.959 to 1.002)	*0.08*	0.994 (0.981 to 1.007)	10.2 (9.2 to 11.3)

[a] level corresponds to the monthly rate per 100,000 children estimated by the model
ED: Emergency department; 95%CI: 95% confidence interval; MH: mental health conditions; SH: self-harm.

© 2024 Valtuille Z et al. *JAMA Network Open.*

eTable 2. Sensitivity Analyses for Monthly Rates of Hospitalizations and Emergency Department (ED) Visits Related to Mental Health Conditions

Model	Pre-pandemic onset period (Jan 2016 – Feb 2020)			Initial pandemic period (March – May 2020)		First year after pandemic onset (June 2020 – May 2021)					Second year after pandemic onset (June 2021 – May 2022)			Third year after pandemic onset (June 2022 – May 2023)			
	**Level Jan. 2016 (95CI)[a]**	**Trend, % (95CI)[b]**	**p-value**	**Change in level, % (95CI)[b]**	**p-value**	**Change in level, % (95CI)[b]**	**p-value**	**Change in trend, % (95CI)[b]**	**p-value**	**Trend, % (95CI)[b]**	**Change in trend, % (95CI)[b]**	**p-value**	**Trend, (95CI)[b]**	**Change in trend, % (95CI)[b]**	**p-value**	**Trend, % (95CI)[b]**	**Level May 2023 [a]**
**Hospitalizations**																	
Log-linear regression adjusted on the total monthly number of hospitalizations	55.8 (50.3 to 61.9)	-0.2 (-0.5 to 0.0)	*0.09*	-9.3 (-14.7 to -3.6)	*0.002*	-4.1 (-11.8 to 4.2)	*0.31*	2.0 (0.6 to 3.4)	*0.005*	1.7 (0.7 to 2.7)	-2.2 (-4.2 to -0.2)	*0.03*	-0.5 (-1.4 to 0.4)	-0.7 (-2.7 to 1.3)	*0.46*	-1.3 (-2.2 to -0.4)	47.2 (42.3 to 52.7)
Negative binomial regression	62.92 (59.36 to 66.7)	0.0 (0.0 to 0.1)	*0.44*	-48.7 (-54.4 to -42.3)	*<0.001*	-12.9 (-22.7 to -1.9)	*0.02*	1.4 (0.2 to 2.7)	*0.02*	1.5 (0.2 to2.7)	-1.9 (-3.5 to -0.3)	*0.02*	-0.5 (-1.0 to 0.0)	-0.4 (-1.3 to 0.4)	*0.33*	-0.9 (1.4 to -0.5)	56.0 (52.7 to 59.4)
**ED visits**																	
Log-linear regression adjusted on the total monthly number of ED visits	32.3 (29.2 to 35.9)	0.3 (0.1 to 0.4)	*0.003*	-44.6 (-50.2 to -38.4)	*<0.001*	-17.6 (-25.7 to -8.6)	*<0.001*	2.6 (1.5 to 3.6)	*<0.001*	2.8 (2.0 to3.5)	-2.9 (-4.5 to -1.4)	*<0.001*	-0.2 (-0.8 to 0.3)	-0.2 (-1.7 to 1.4)	*0.82*	-0.4 (-1.1 to 0.2)	46.7 (43.2 to 50.2)
Negative binomial regression	44.15 (42.79 to 45.56)	0.3 (0.2 to 0.4)	*<0.001*	-52.7 (-55.8 to -49.3)	*<0.001*	-18.8 (-26.8 to -10.1)	*<0.001*	2.3 (1.1 to 3.5)	*<0.001*	2.6 (1.4 to 3.8)	-2.4 (-4.2 to -0.5)	*0.01*	0.1 (-0.8 to 1.1)	-0.9 (-2.5 to 0.8)	*0.28*	-0.8 (-1.6 to 0.1)	53.7 (50.8 to 56.8)

[a] level corresponds to the monthly rate per 100,000 children and adolescents estimated by the model
[b] Model estimates for trend and changes in level and trend are presented as percentage changes per month (95%CI).
ED: emergency department, 95%CI: 95% confidence interval

© 2024 Valtuille Z et al. *JAMA Network Open.*

eTable 3. Sensitivity Analyses for Monthly Rates of Hospitalizations and Emergency Department (ED) Visits Related to Self-Harm

Model	Pre-pandemic onset period (Jan 2016 – Feb 2020)			Initial pandemic period (March – May 2020)		First year after pandemic onset (June 2020 – May 2021)					Second year after pandemic onset (June 2021 – May 2022)			Third year after pandemic onset (June 2022 – May 2023)			
	Level Jan. 2016 (95CI)[a]	Trend, % (95CI)[b]	p-value	Change in level, % (95CI)[b]	p-value	Change in level, % (95CI)[b]	p-value	Change in trend, % (95CI)[b]	p-value	Trend, (95CI)[b]	Change in trend, % (95CI)[b]	p-value	Trend, % (95CI)[b]	Change in trend, % (95CI)[b]	p-value	Trend, % (95CI)[b]	Level May 2023 [a]
**Hospitalizations**																	
Log-linear regression adjusted on the total monthly number of hospitalizations	8.8 (7.7 to 10.0)	0.2 (0.1 to 0.4)	*0.003*	-33.6 (-40.5 to -25.8)	*<0.001*	-19.7 (-27.4 to -11.3)	*<0.001*	4.6 (3.6 to 5.7)	*<0.001*	4.9 (3.7 to 6.0)	-4.3 (-5.9 to -2.7)	*<0.001*	0.4 (-0.5 to 1.3)	-1.5 (-3.0 to 0.1)	*0.06*	-1.1 (-2.1 to -0.1)	14.5 (13.5 to 15.6)
Negative binomial regression	12.3 (11.6 to 13.1)	0.3 (0.2 to 0.4)	*<0.001*	-43.9 (-49.0 to -38.3)	*<0.001*	-21.0 (-28.0 to -13.3)	*<0.001*	4.5 (3.4 to 5.5)	*<0.001*	4.8 (3.8 to 5.8)	-4.1 (-5.5 to -2.7)	*<0.001*	0.5 (-0.2 to 1.2)	-1.7 (-3.0 to -0.3)	*0.02*	-1.2 (-2.0 to -0.4)	18.1 (16.9 to 19.3)
Stringent definition of SH	6.8 (6.6 to 7.0)	0.1 (0.0 to 0.2)	*0.09*	-41.9 (-45.8 to -37.7)	*<0.001*	-22.9 (-28.2 to -17.2)	*<0.001*	4.6 (3.8 to 5.4)	*<0.001*	4.7 (3.9 to 5.5)	-3.5 (-4.6 to -2.3)	*<0.001*	1.1 (0.5 to 1.7)	-2.5 (-3.6 to -1.3)	*<0.001*	-1.5 (-2.1 to -0.8)	9.1 (8.6 to 9.6)
**ED visits**																	
Log-linear regression adjusted on the total monthly number of ED visits	5.7 (5.0 to 6.6)	0.7 (0.5 to 0.9)	*<0.001*	-36.6 (-44.6 to -27.5)	*<0.001*	-13.5 (-24.3 to -1.1)	*0.03*	4.1 (2.7 to 5.5)	*<0.001*	4.8 (3.7 to 6.0)	-3.1 (-5.2 to -1.0)	*0.004*	1.5 (0.7 to 2.4)	-1.9 (-3.9 to 0.1)	*0.06*	-0.4 (-1.4 to 0.5)	16.5 (14.9 to 18.2)
Negative binomial regression	8.2 (7.8 to 8.6)	0.8 (0.6 to 0.9)	*<0.001*	-45.6 (-51.5 to -38.9)	*<0.001*	-16.6 (-25.9 to -6.1)	*0.003*	4.0 (2.8 to 5.2)	*<0.001*	4.8 (3.5 to 6.0)	-2.8 (-4.5 to -1.1)	*0.001*	1.8 (1.0 to 2.6)	-2.5 (-3.8 to -1.2)	*<0.001*	-0.8 (-1.5 to -0.1)	19.4 (18.2 to 20.7)
Stringent definition of SH	0.5 (0.5 to 0.6)	-0.4 (-0.8 to 0.0)	*0.03*	-29.0 (-42.9 to -11.8)	*0.002*	-15.4 (-34.0 to 8.3)	*0.18*	6.4 (3.7 to 9.3)	*<0.001*	6.0 (3.3 to 8.7)	-6.7 (-10.6 to -2.7)	*0.002*	-1.1 (-3.2 to 0.9)	-3.4 (-7.2 to 0.5)	*0.08*	-4.5 (-6.7 to -2.3)	0.4 (0.3 to 0.4)

[a] level corresponds to the monthly rate per 100,000 children and adolescents estimated by the model
[b] Model estimates for trend and changes in level and trend are presented as percentage changes per month (95%CI).
ED: emergency department, 95%CI: 95% confidence interval

© 2024 Valtuille Z et al. *JAMA Network Open.*

eFigure 1. Changes in Rates and Trends of Mental Health Hospitalizations and ED Visits in Girls and Boys Aged 6 to 11 Years in France

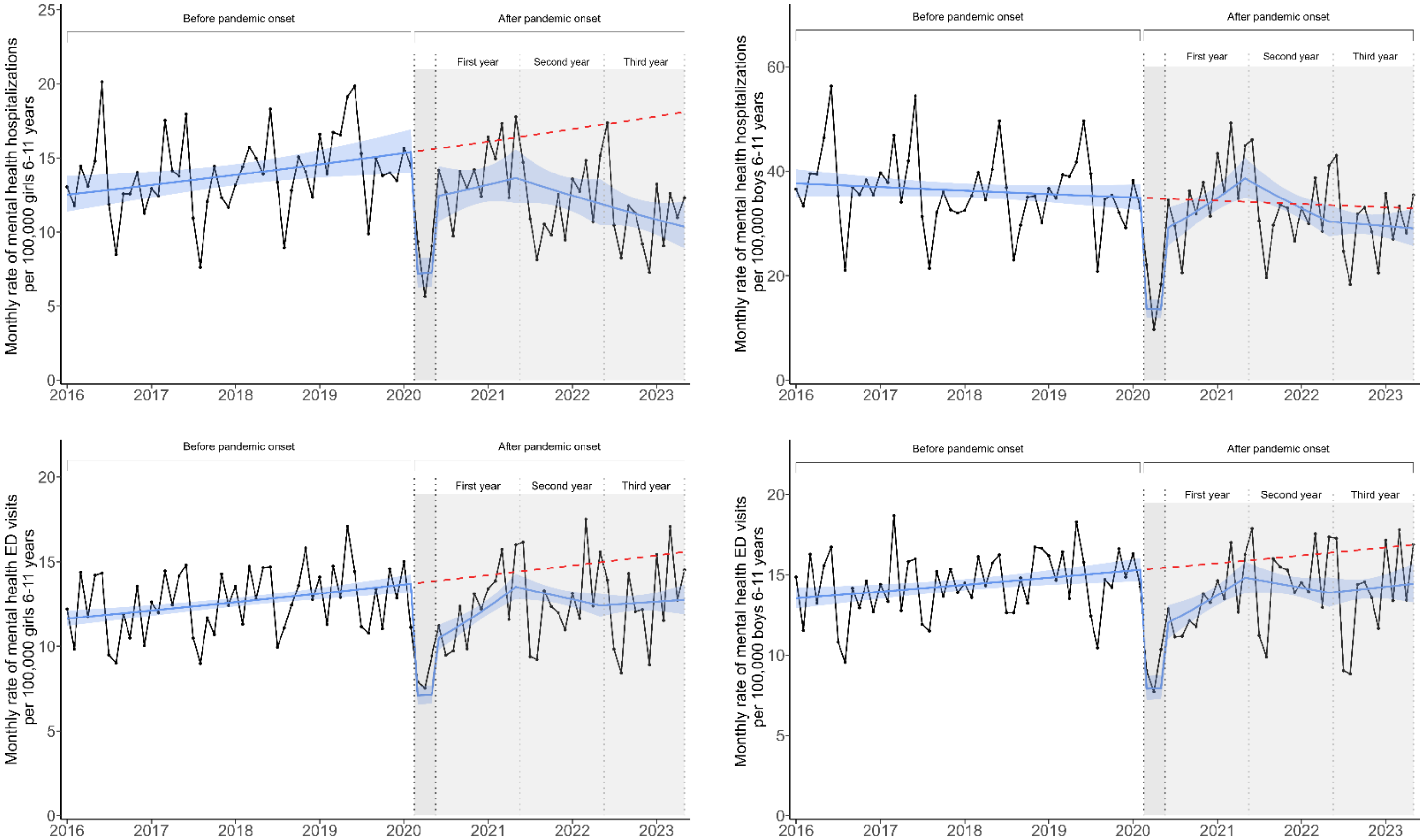

Bold black lines indicate the observed mental health hospitalization or ED visit rates per 100,000 girls 6-11 years (left) and boys 6-11 years (right). Bold blue lines indicate rates estimated by the model with corresponding 95%CIs. The red dotted lines indicate the expected rates based on pre-pandemic data. Before the pandemic onset: January 2016 to February 2020. Initial pandemic period: March to May 2020.

First year after pandemic onset: June 2020 to May 2021; Second year after pandemic onset: June 2021 to May 2022; Third year after pandemic onset: June 2022 to May 2023.

ED: Emergency department; 95%CI: 95% confidence interval; MH: mental health conditions.

© 2024 Valtuille Z et al. *JAMA Network Open.*

eFigure 2. Changes in Rates and Trends of Mental Health Hospitalizations and ED Visits in Girls and Boys Aged 12 to 17 Years in France

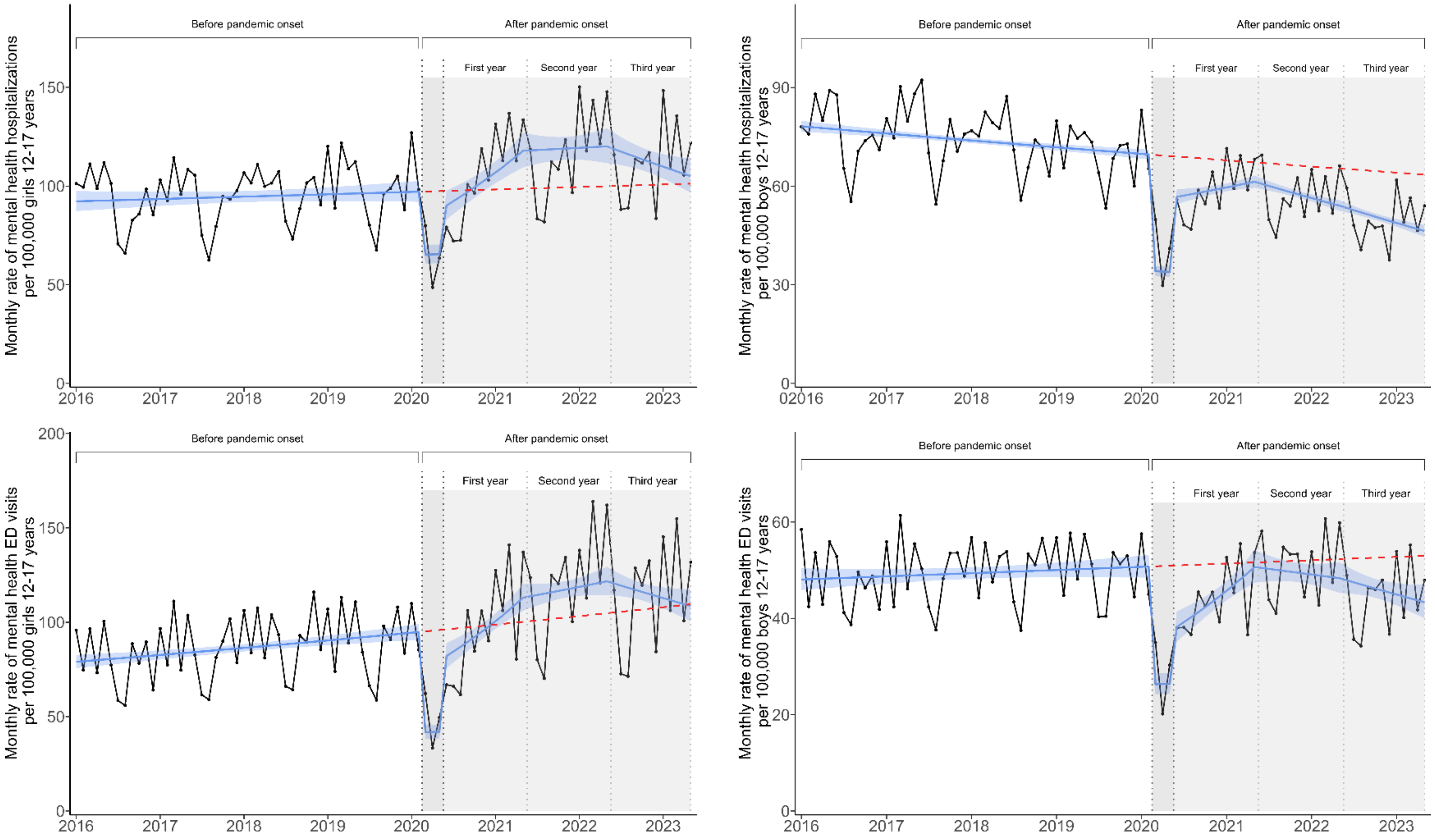

Bold black lines indicate the observed mental health hospitalization or ED visit rates per 100,000 girls 12-17 years (left) and boys 12-17 years (right). Bold blue lines indicate rates estimated by the model with corresponding 95%CIs. The red dotted lines indicate the expected rates based on pre-pandemic data. Before the pandemic onset: January 2016 to February 2020. Initial pandemic period: March to May 2020.

First year after pandemic onset: June 2020 to May 2021; Second year after pandemic onset: June 2021 to May 2022; Third year after pandemic onset: June 2022 to May 2023.

ED: Emergency department; 95%CI: 95% confidence interval; MH: mental health conditions.

© 2024 Valtuille Z et al. *JAMA Network Open.*

eFigure 3. Changes in Rates and Trends of Self-Harm–Related Hospitalizations and ED Visits in Girls and Boys Aged 6 to 11 Years in France

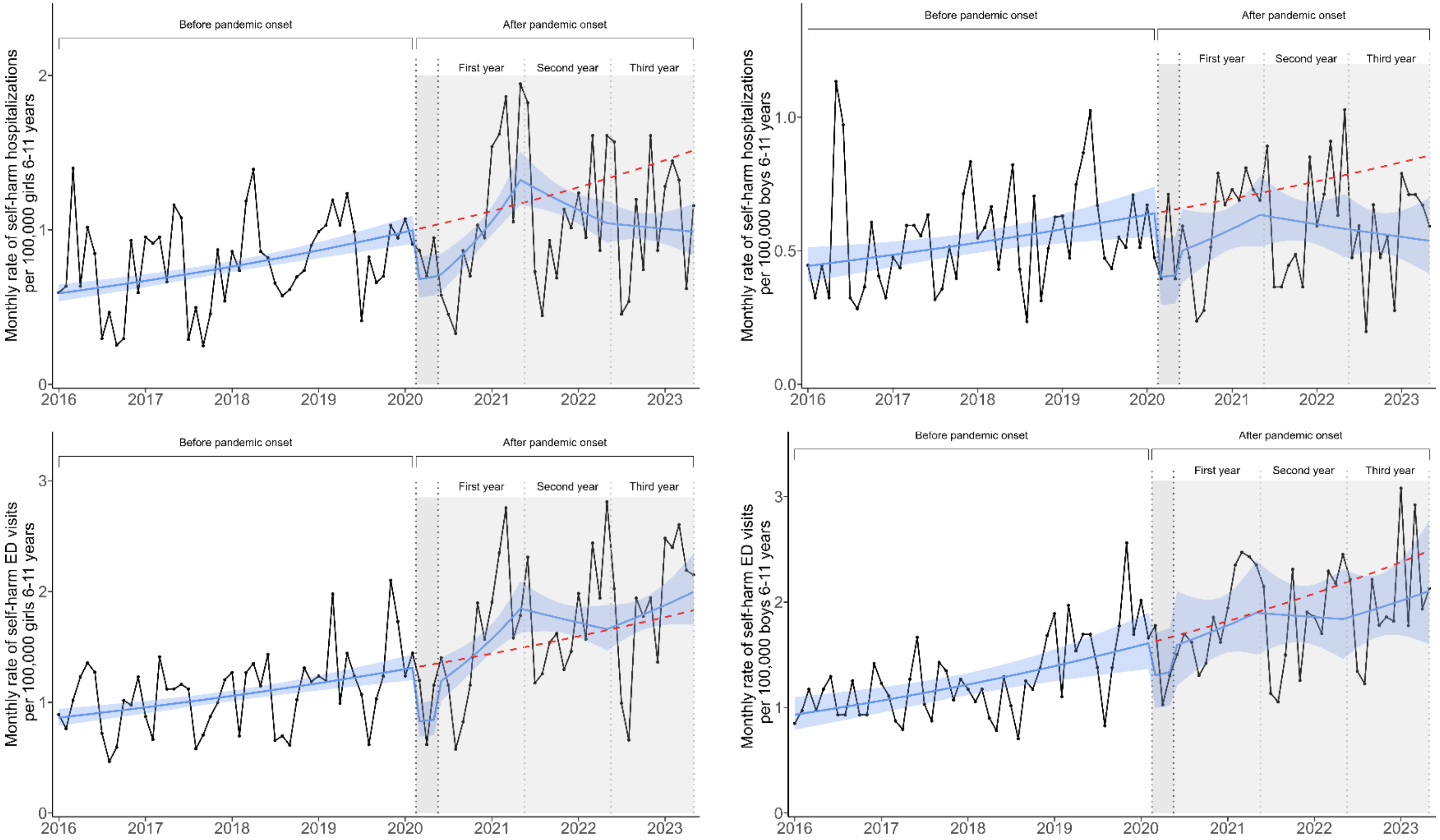

Bold black lines indicate the observed self-harm related hospitalization or ED visit rates per 100,000 girls 6-11 years (left) and boys 6-11 years (right). Bold blue lines indicate rates estimated by the model with corresponding 95%CIs. The red dotted lines indicate the expected rates based on pre-pandemic data. Before the pandemic onset: January 2016 to February 2020. Initial pandemic period: March to May 2020.

First year after pandemic onset: June 2020 to May 2021; Second year after pandemic onset: June 2021 to May 2022; Third year after pandemic onset: June 2022 to May 2023

ED: Emergency department; 95%CI: 95% confidence interval; SH: self-harm.

© 2024 Valtuille Z et al. *JAMA Network Open.*

eFigure 4. Changes in Rates and Trends of Self-Harm–Related Hospitalizations and ED Visits in Girls and Boys Aged 12 to 17 Years In France

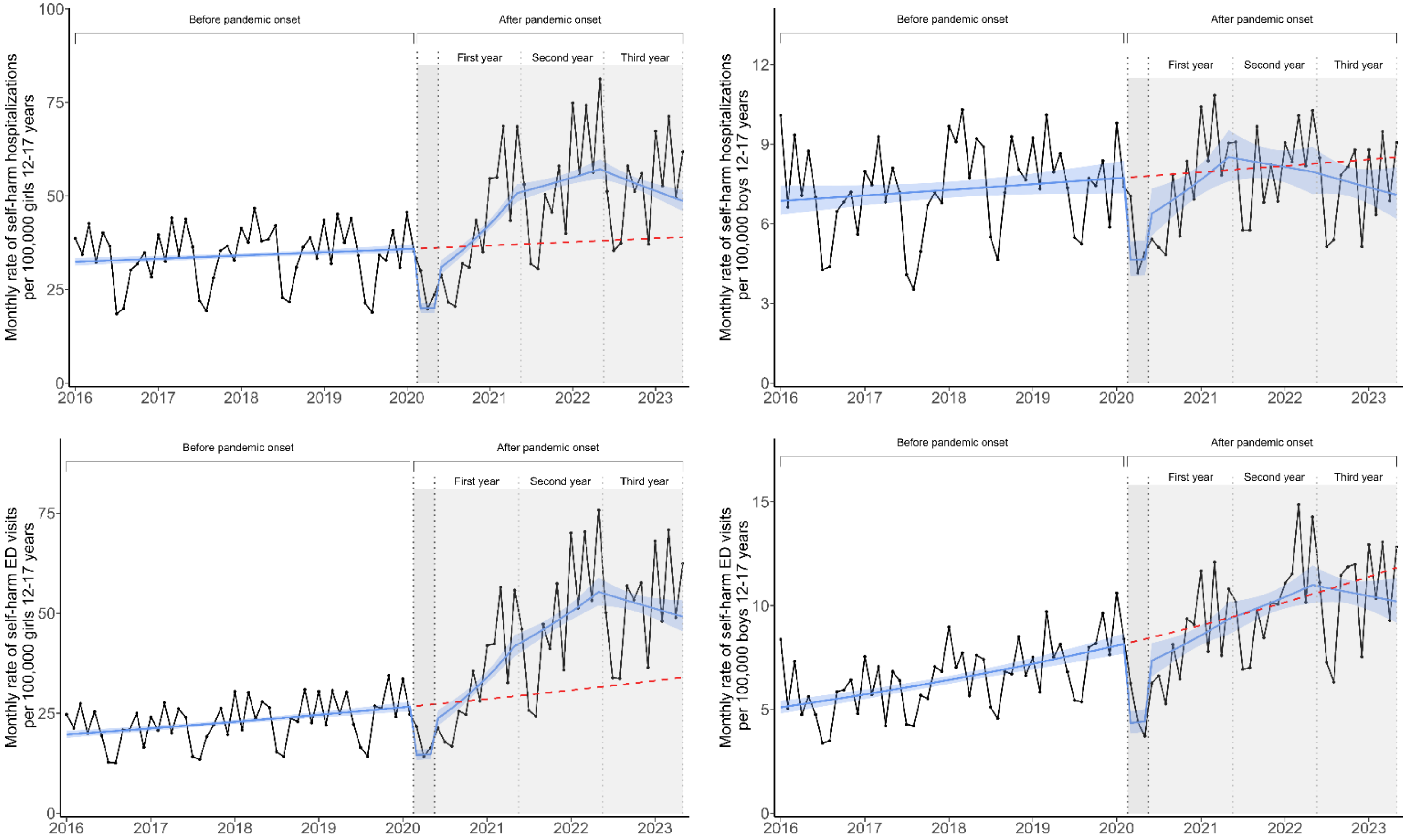

Bold black lines indicate the observed self-harm related hospitalization or ED visit rates per 100,000 girls 12-17 years (left) and boys 12-17 years (right). Bold blue lines indicate rates estimated by the model with corresponding 95%CIs. The red dotted lines indicate the expected rates based on pre-pandemic data. Before the pandemic onset: January 2016 to February 2020. Initial pandemic period: March to May 2020.

First year after pandemic onset: June 2020 to May 2021; Second year after pandemic onset: June 2021 to May 2022; Third year after pandemic onset: June 2022 to May 2023

ED: Emergency department; 95%CI: 95% confidence interval; SH: self-harm.

© 2024 Valtuille Z et al. *JAMA Network Open.*

eFigure 5. Correlograms and Residual Analysis of Primary Analyses

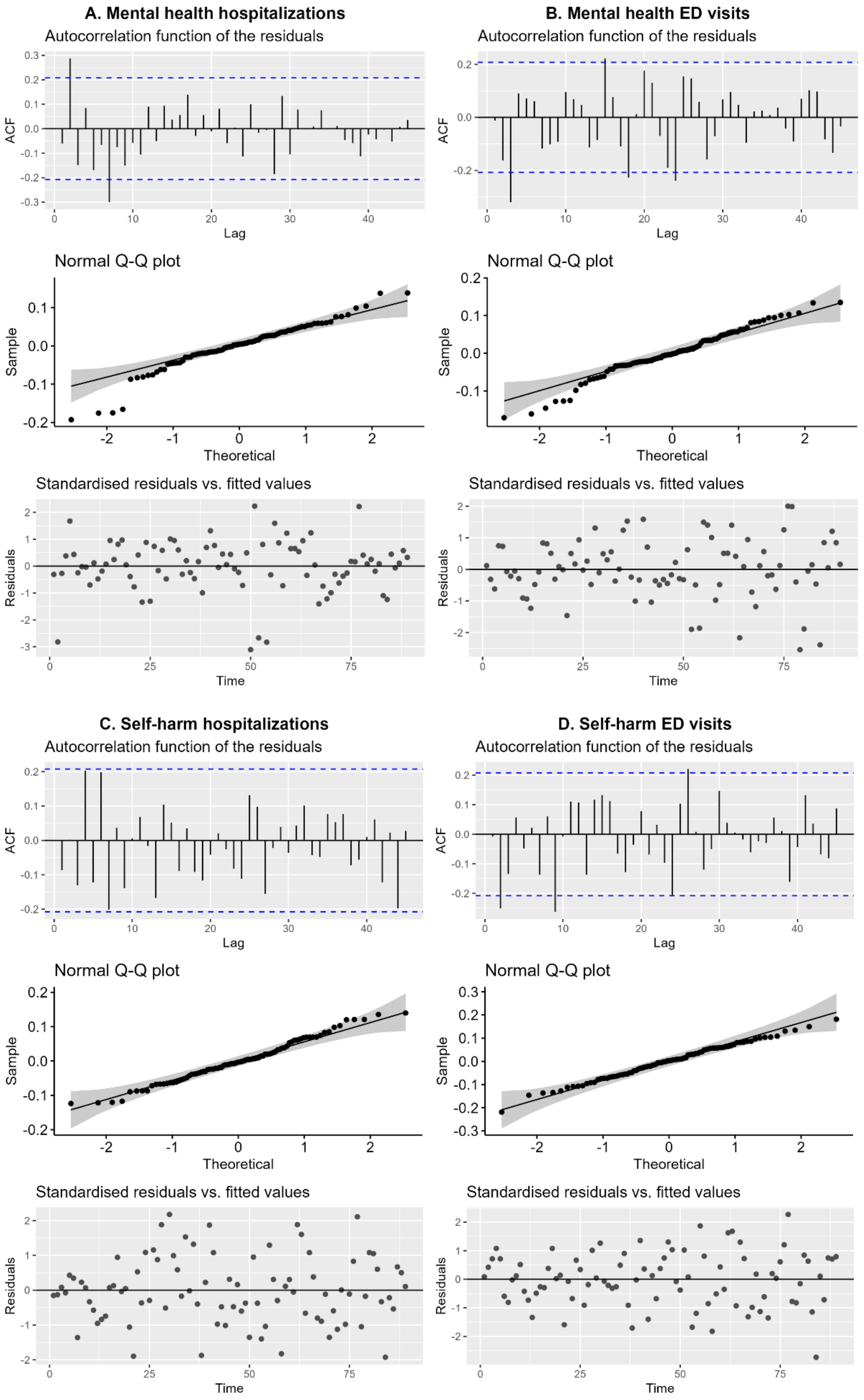

Abbreviations: ACF: autocorrelation function- RQR: randomized quantile residuals- ED: emergency department

© 2024 Valtuille Z et al. *JAMA Network Open.*

eFigure 6. Correlograms and Residual Analysis of Sensitivity Analyses: Mental Health

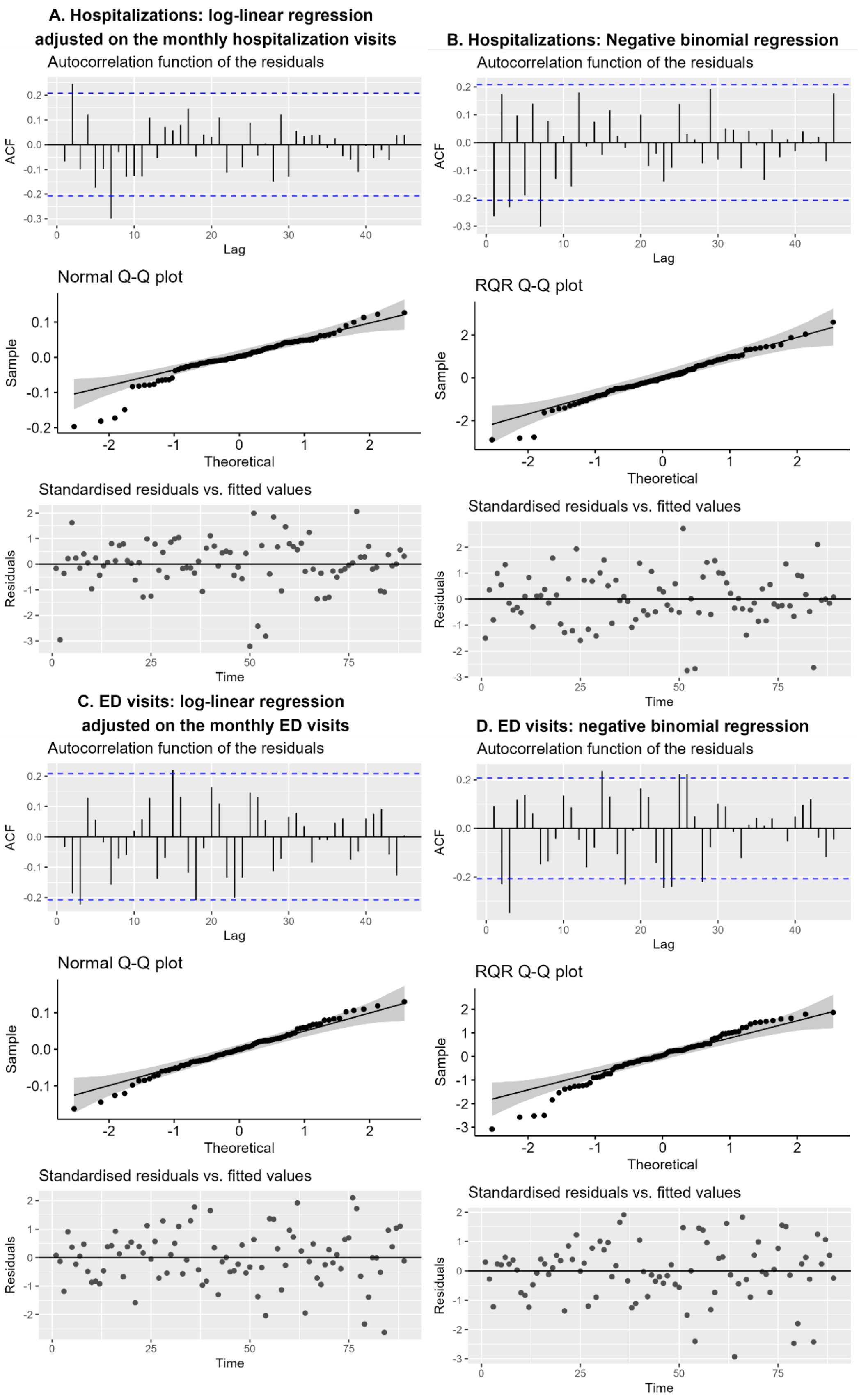

Abbreviations: ACF: autocorrelation function; RQR: randomized quantile residuals; ED: emergency department

© 2024 Valtuille Z et al. *JAMA Network Open.*

eFigure 7. Correlograms and Residual Analysis of Sensitivity Analyses: Self-Harm

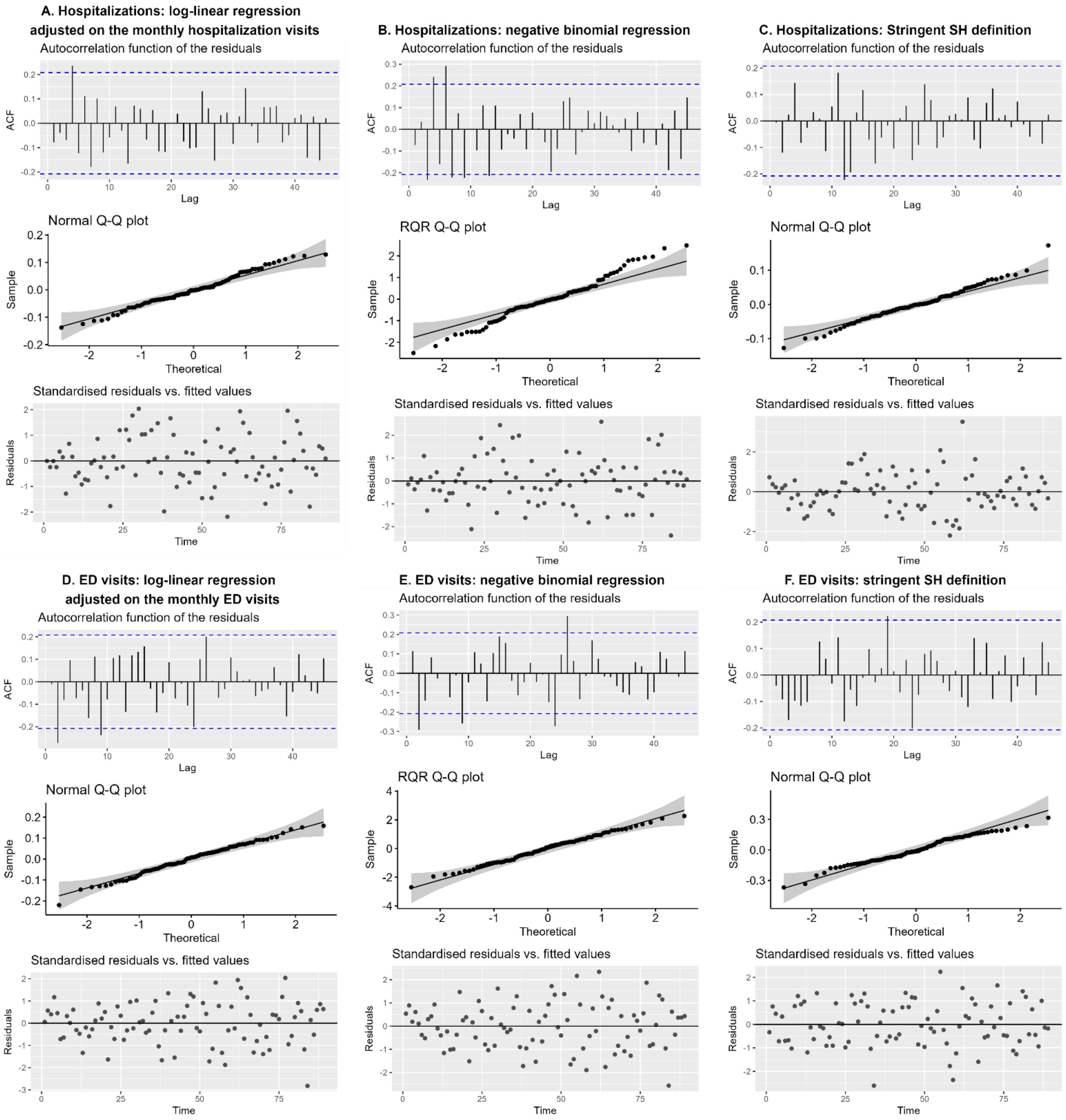

Abbreviations: ACF: autocorrelation function; RQR: randomized quantile residuals; ED: emergency department; SH: self-harm.

© 2024 Valtuille Z et al. *JAMA Network Open.*
