# Supplementary material for: Pediatric Hospitalizations and Emergency Department Visits Related to Mental Health Conditions and Self-Harm
Source: JAMA Netw Open. 2024 Oct 29;7(10):e2441874. doi: 10.1001/jamanetworkopen.2024.41874 (PMC11522941; doi:10.1001/jamanetworkopen.2024.41874)
Supplement: Supplement 2. — Data Sharing Statement [file jamanetwopen-e2441874-s002.pdf]

Data Sharing Statement

Valtuille. Pediatric Hospitalizations and Emergency Department Visits Related to Mental Health Conditions and Self-Harm. *JAMA Netw Open*. Published October 29, 2024. doi:10.1001/jamanetworkopen.2024.41874

Data

Data available: The clinical codes, data management and analysis code used in this study are available on request from the corresponding author. Access to the data dictionary and these anonymized data can be granted upon request to the ATIH platform. The criteria for applying for these data are available in the ATIH website: https://www.atih.sante.fr/acces-aux-donnees-pour-les-etablissements-de-sante-les-chercheurs-et-les-institutionnels.
